# Hydrogen From Simulated Seawater Using Long‐Term Stable and Cost‐Effective Plasmonic Photocatalysts

**DOI:** 10.1002/cssc.71072

**Published:** 2026-09-22

**Authors:** Fons Dingenen, Manu Donders, Rajeshreddy Ninakanti, Christine Vantomme, Daniel Arenas‐Esteban, Rituraj Borah, Sara Bals, Sammy W. Verbruggen

**Affiliations:** ^1^ Antwerp Engineering Photoelectrochemistry and Sensing (A‐PECS) Department of Bioscience Engineering University of Antwerp Antwerp Belgium; ^2^ NANOlight Center of Excellence University of Antwerp Antwerp Belgium; ^3^ Electron Microscopy for Material Science (EMAT) Department of Physics University of Antwerp Antwerp Belgium

**Keywords:** bimetallic nanoparticle, core‐shell nanoparticle, hydrogen, layer by layer, photocatalysis, photocatalytic water splitting, plasmonic nanoparticles, polyaniline, seawater, surface plasmon resonance

## Abstract

Generating hydrogen directly from seawater is an attractive feature for realizing large‐scale solar fuel production. Here, we present an effective photocatalyst design that not only considers solar light activity but also long‐term durability and optimized manufacturing costs. A series of stabilized bimetallic Au–Ag plasmonic “rainbow” nanoparticles were synthesized and grafted at low loadings onto TiO_2_ substrates, yielding strong visible‐light absorption and enhanced photocatalytic activity. To address the intrinsic instability of (plasmonic) metal nanoparticles in saline environments, two encapsulation strategies were investigated: insulating layer‐by‐layer (LbL) polyelectrolyte shells and conductive polyaniline (PANI) shells formed via in situ polymerization. In simulated seawater, a maximum H_2_ evolution rate of 348 ± 108 µmol g^−1^ h^−1^ was achieved for TiO_2_ + 2 wt% “rainbow” PANI‐stabilized nanoparticles. PANI‐stabilized plasmonic catalysts outperformed both bare and LbL‐coated samples while maintaining full activity even after 1 month of storage in the dark in simulated seawater. These results demonstrate that conductive polymer encapsulation effectively preserves plasmonic activity even in harsh media, offering a prospect toward durable and economically viable solar hydrogen production from simulated seawater.

## Graphical abstract

Can plasmonic photocatalysts withstand a saline environment? Yes! A conductive polyaniline shell stabilizes gold–silver nanoparticles while largely preserving charge transfer to TiO_2_, achieving 348 ± 108 μmol H_2_ g^−1^ h^−1^. Moreover, the catalyst survived 1 month of storage in simulated seawater, with almost full retention of its initial activity. This encapsulation strategy is shown to enable cost‐effective solar hydrogen production.

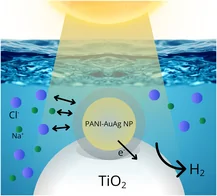

**Abbreviations**
ACER: average cost‐effectiveness ratio
DEI: direct electron injection
HOV: hydrogen overvoltage
IC: ion chromatography
ICER: incremental cost‐effectiveness ratio
LbL: layer‐by‐layer
NFE: near‐field enhancement
NP(s): nanoparticle(s)
PDD: pulsed discharge detector
SDS: sodium dodecyl sulfate
STH: solar‐to‐hydrogen (efficiency)
TCD: thermal conductivity detector.

## 1 Introduction

The accelerating depletion of fossil fuels and their associated greenhouse emissions demand a transition toward sustainable, carbon‐neutral energy carriers. Hydrogen offers a viable solution because it can store intermittent renewable energy and releases only water vapor upon use [1, 2]. However, most industrially produced hydrogen is still derived from fossil sources, motivating solar‐driven water splitting as a clean alternative [3]. Since the pioneering work of Fujishima and Honda, advances in photocatalyst design have greatly improved light harvesting and charge separation, yet practical applications remain limited by low solar‐to‐hydrogen (STH) efficiencies and poor stability under realistic conditions [4]. Nonetheless, some major breakthroughs have been made over the past decade. Pinaudet al. calculated that the price for photocatalytically produced H_2_ could drop well below the US DOE target of 2–4 $ kg^−1^ if STH efficiencies above 10% are reached [5]. Recent systems have achieved STH efficiencies above 1%, such as sheet‐type SrTiO_3_ panels that maintain long‐term stability but still require purified water and UV excitation [6, 7]. Since freshwater represents less than 3% of the Earth’s accessible water reserves, direct seawater splitting is even more attractive for large‐scale hydrogen production [3, 8, 9]. Unfortunately, the high ionic strength and chloride content in seawater promote Cl_2_ evolution, surface corrosion, and nanoparticle (NP) aggregation, while Mg^2+^ and Ca^2+^ salts cause scaling and catalyst surface passivation [9]. Zhou et al. achieved 9.2% STH in pure water using InGaN/GaN nanowires decorated with Rh@Cr_2_O_3_ and Co_3_O_4_ cocatalysts, but the efficiency dropped to 6.2% in seawater due to rapid catalyst degradation [10]. Plasmonic photocatalysis is another promising strategy for improving the solar light response [11, 12, 13, 14]. Here, plasmonic materials, such as Au–Ag–semiconductor hybrids, broaden solar absorption and enhance charge separation, with recent advances further optimizing photocatalytic hydrogen evolution through alloy engineering and interface design [15]. Recent work by An et al. demonstrated high H_2_ evolution activity of plasmonic Au/ZnIn_2_S_4_/WO_3_ in simulated seawater, together with good stability during photocatalytic cycling [16]. Nevertheless, long‐term stability in saline environments remains an important challenge, particularly for metallic components susceptible to halide corrosion [17, 18].

To address these issues, researchers increasingly rely on interface engineering and particle encapsulation but with limited success so far. For instance, Xia et al. reported on TiO_2_/reduced graphene oxide/Cu composites exploiting the electron sink effect, yet the observed activity decreased by 35.5% after six cycles in simulated seawater, highlighting the persistent challenge of stability in saline media [19].

In our previous work, we have already demonstrated that TiO_2_ modified with plasmonic Au–Ag/polymer core–shell nanoparticles shows excellent optical tunability and retains its activity during harsh oxidative degradation reactions [18]. The shell provided nanoscale control over thickness and markedly improved nanoparticle stability without suppressing near‐field enhancement (NFE). However, the encapsulation strategy that was employed is based on a layer‐by‐layer (LbL) approach that only yields insulating shells through a tedious, hard‐to‐scale, and labor‐intensive process. In this work, we have therefore explored in situ polymerization of aniline to polyaniline (PANI) as a capping strategy [20]. This allowed for a synthetic strategy that is faster, easier to scale, and yields conductive shells that enable electron transfer mechanisms across its interface. By combining broadband plasmonic enhancement by bimetallic NPs with conductive polymer shell stabilization, this study aims to develop cost‐effective and chemically robust plasmonic photocatalysts for hydrogen production directly from simulated seawater.

## 2 Materials and Methods

### 2.1 Synthesis and Characterization of Plasmonic “Rainbow” Photocatalyst

The plasmonic “rainbow” photocatalyst was synthesized following previously established procedures in our group [17, 18]. Briefly, two colloidal spherical Au_*x*_Ag_1−*x*_ nanoparticle suspensions (*x*  =  0.3 and 0.7) were synthesized using a modified Turkevich method [17, 21]. Diluted 10 mM HAuCl_4_.3 H_2_O (Sigma‐Aldrich, > 99.9%) and 10 mM AgNO_3_ (Sigma‐Aldrich, >99%) precursor solutions were mixed to obtain a total metal concentration of 0.1 mM, heated to boil under vigorous stirring, and reduced with 1 mL of freshly prepared 1 wt% sodium citrate solution (Sigma‐Aldrich, 99%). After 30 min, reactions were quenched in an ice bath. NP formation was confirmed by UV–vis spectroscopy (Shimadzu UV–vis 2501 PC). Equal volumes of both suspensions were combined to form the “rainbow” mixture.

Subsequently, P25 TiO_2_ (ACROS Organics, ≥99.5%, ~80% anatase, ~20% rutile) was modified via photo‐impregnation to yield “rainbow” photocatalysts with nominal loadings in the range from 0 to 5 wt%. The plasmonic photocatalysts were characterized by N_2_ sorption (Brunauer–Emmett–Teller (BET), Micromeritics TriStar 3000 V6.04A; degassed overnight at 200 °C), diffuse reflectance spectroscopy (DRS) (Shimadzu UV–vis 2501 PC with 60 mm BaSO_4_‐coated integrating sphere; 1.5 g BaSO_4_ as carrier and blank; bandgaps estimated via Kubelka–Munk transformation and Tauc analysis), and X‐ray diffraction (Bruker D8 Advance, Cu Kα = 1.54 Å, 40 kV, 40 mA, 2*θ* = 20°–80°, 0.5 s step^−^^1^).

### 2.2 Synthesis and Characterization of Layer‐by‐Layer‐ and Polyaniline‐Stabilized Plasmonic Photocatalyst

Stabilization was achieved via two methods: the LbL approach, described previously [18], and in situ PANI polymerization, adapted for Au_*x*_Ag_1−*x*_ hybrids [20, 22].

For the latter, 72 mL NP suspensions were concentrated via centrifugation into ca. 1.2 mL in demineralized water and then added dropwise to a premixed solution of 27 mL 2 mM aniline (Sigma‐Aldrich, for synthesis) and 5.4 mL 40 mM sodium dodecyl sulfate (SDS) (≥99.0%). After vortex mixing (5 s), this was added to 27 mL 2 mM ammonium persulfate (≥98.0%) in 10 mM HCl. The mixture was acidified to pH < 2 with 1 M HCl, vortexed again (5 s), and left to react in darkness without agitation. After 0.5, 1, 2, 3, and 24 h, Au_*x*_Ag_1−*x*_@PANI NPs were collected, centrifuged, washed, and stored in 4 mM SDS to prevent aggregation [20]. UV–vis spectroscopy confirmed that the particles demonstrated a redshift, indicative of progressive shell growth over time. The final metal concentrations were quantified by Spectroquant elemental analysis (NOVA 60, Merck 114 821 test kit).

For PANI‐coated Au_*x*_Ag_1−*x*_ nanoparticles, a nanoparticle suspension (3 μL) was drop‐cast on a Mo grid with a single layer of graphene and left to dry in ambient air. Bright‐field transmission electron microscopy (BF‐TEM) was performed to visualize the polymer layer around Au nanoparticle. For Au_*x*_Ag_1−*x*_ nanoparticles deposited on TiO_2_, a nanoparticle suspension (3 μL) was drop‐cast on a Cu‐supported carbon grid and left to dry in ambient air. High‐angle annular dark‐field scanning transmission electron microscopy (HAADF–STEM) and energy‐dispersive X‐ray spectroscopy (EDXS) were performed to understand the distribution of Au–Ag nanoparticles on TiO_2_. Both analyses were performed using a Thermo Fischer Osiris microscope operated at 200 kV. Stabilized NPs were deposited on P25 TiO_2_ by photo‐impregnation, as done for bare bimetallic NPs. Modified photocatalysts were further characterized by DRS, BET, and their final metal loading was determined by EDXS.

### 2.3 Stability Testing

To evaluate nanoparticle stability, two tests were performed: a hot air treatment and a salt addition test. For the hot air treatment, concentrated suspensions of both the original and PANI‐stabilized NPs were drop‐casted onto pre‐cleaned glass substrates and heated in air at 100 °C for 24 h [18]. For the salt addition test, 150 µL of a 1 M NaCl solution (Sigma‐Aldrich, ≥99.0%) was added to 1.5 mL of each NP suspension (bare and PANI‐stabilized). All samples were adjusted to contain equal concentrations based on the Spectroquant quantification. UV–vis absorbance spectra were recorded before and after salt addition using a Shimadzu UV–vis 2501 PC double‐beam spectrophotometer. The results were compared with LbL stabilization data acquired previously [18].

### 2.4 XTT Test

To monitor the oxygen activation efficiency (O_2_^−^ formation) by plasmonic metal NPs, 2,3‐bis(2‐methoxy‐4‐nitro‐5‐sulfophenyl)‐2H‐tetrazolium‐5‐carboxanilide (XTT) was used as a probe molecule as it reacts with superoxide anions to form formazan. As such, XTT to formazan conversion provides a semiquantitative proxy for electron transfer‐dependent oxygen activation. Four samples were evaluated by the XTT assay: (i) bare plasmonic “rainbow” nanoparticles, (ii) PANI‐coated “rainbow” nanoparticles, (iii) bare Au_0.3_Ag_0.7_ nanoparticles, and (iv) Au_0.__3_Ag_0.__7_ nanoparticles coated with four polyelectrolyte layers (LbL strategy). A single‐particle composition was used for the latter for time efficiency reasons without compromising the interpretation. Each sample was deposited onto amine‐functionalized SiO_2_. After washing and drying overnight in a vacuum oven (35 °C), 5 mg of photocatalyst powder was suspended in a XTT sodium salt solution (10 mL, 0.1 mM, Sigma‐Aldrich, ≥90%) in DMSO (dimethyl sulfoxide, Emplura, 99.0%) in a 10‐mL glass vial. The resulting suspension was purged with oxygen (5 min, 50 mL min^−1^) to ensure oxygen saturation. The glass vial was subsequently illuminated using AM 1.5G simulated sunlight (SciSun‐300, Sciencetech) with an intensity of 100 mW/cm [2]. After 1 h, 1.5 mL of the suspension was centrifuged at 12.4 g and the resulting supernatant was analyzed by UV–vis (Shimadzu UV–vis 2501 PC) to measure the formation of formazan at *λ*_max_ = 480 nm.

### 2.5 Photocatalytic H_2_ Production Experiments

Photocatalytic hydrogen production experiments in (simulated sea)water were performed in a batch reactor, in which a slurry of 10 mg photocatalyst in 40 mL of a water/methanol (Chem‐Lab, ≥99.0%) (v/v 7:1) mixture was placed in a Teflon liner (Figure S1). Prior to irradiation, the reactor headspace was purged with N_2_ overnight to remove residual oxygen. The temperature was regulated by a cooling mantle and monitored with a thermocouple; only a minor increase from 19 to 21 °C was observed under illumination (Figure S2). Irradiation was performed through a circular quartz window (5 cm diameter) using AM 1.5G simulated sunlight (SciSun‐300, Sciencetech) at an intensity of 100 mW cm^−2^ (1 sun), measured at the bottom of the reactor and calibrated between 300 and 1100 nm with a spectroradiometer (Avantes Avaspec‐3648‐USB2). Gas samples (2 mL) were periodically withdrawn from the headspace using a syringe fitted with a 12‐cm needle inserted into a tilted inlet. The evolved gases were analyzed by gas chromatography (CompactGC 4.0, Interscience) equipped with a pulsed discharge detector (detection limit ≈ 1 ppm) for the low initial concentrations and a thermal conductivity detector for final higher concentrations. Sampling intervals were adjusted according to photocatalyst activity.

For simulated seawater measurements, a 0.5 M NaCl (Sigma‐Aldrich, >99.0%) solution was used. The stability of the photocatalysts was determined via storage aging and photocatalytic cycling experiments. The aging experiments consisted of retesting the sample after 1 month of storage, during which the samples were kept in simulated seawater (0.5 M NaCl) on the shelf in the dark. Stability was also studied via four consecutive 4 h illumination cycles (16 h total), with the reaction medium replaced with fresh simulated seawater after each cycle. The salt content was verified using ion chromatography (IC), followed by anion and cation ion conductivity detectors using a Metrohm 930 Compact IC Flex and Eco IC, respectively. The loss of Cl^−^ ions was measured before and after the H_2_ evolution measurement, to ascertain if any Cl_2_ evolution or Cl^−^ adsorption had occurred.

### 2.6 COMSOL Simulations

Near‐field enhancement of the plasmonic nanoparticles was studied by finite element analysis using the wave optics module in COMSOL Multiphysics. To represent the metal nanoparticle, a 3D sphere of 15 nm in diameter was built and a shell of 5 nm was added around the cores to represent the PANI shell. The PANI domain was meshed using a predefined mesh with normal mesh size. Air is taken as the surrounding medium and a plane wave polarized in the *X*‐axis direction and propagating along the *Y*‐axis was used for solving the scattered field of Maxwell’s wave equations in a wavelength domain study. The wavelength of the incident wave with a magnitude of *E*_0_  = 1 V/m depends on the overall nanoparticle structure. In the wave optics module of the COMSOL Multiphysics program, Maxwell’s electromagnetic wave equation is solved for the scattered electric field. The wave‐dependent refractive index of the metal alloys and conducting polymer shell is taken from the literature [23, 24].

## 3 Results and Discussion

### 3.1 Optical Characterization of Plasmonic “Rainbow” Photocatalyst

**FIGURE 1:**
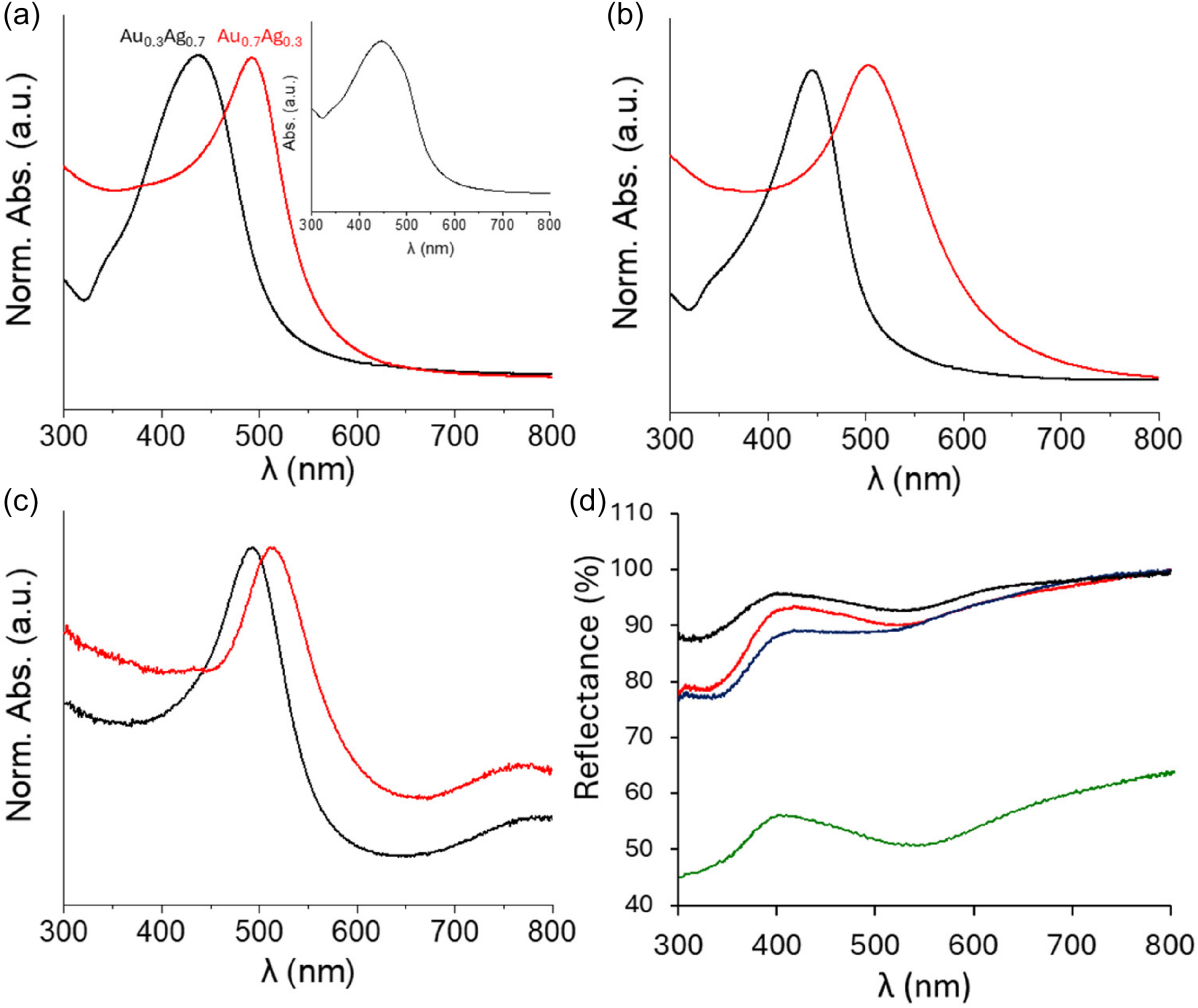
UV–vis spectra of (a) bare (inset: broadband combination), (b) LbL‐, and (c) PANI‐stabilized Au_0.3_Ag_0.7_ and Au_0.7_Ag_0.3_ NP suspensions. Panel (d) shows DRS spectra for P25 + 2 wt% nine‐component rainbow (black, based on data from Dingenen et al. 2021) [18] and P25 + 2 wt% non‐ (red), PANI‐ (green), and LbL‐stabilized (blue) two‐component “rainbow” NPs.

**FIGURE 2:**
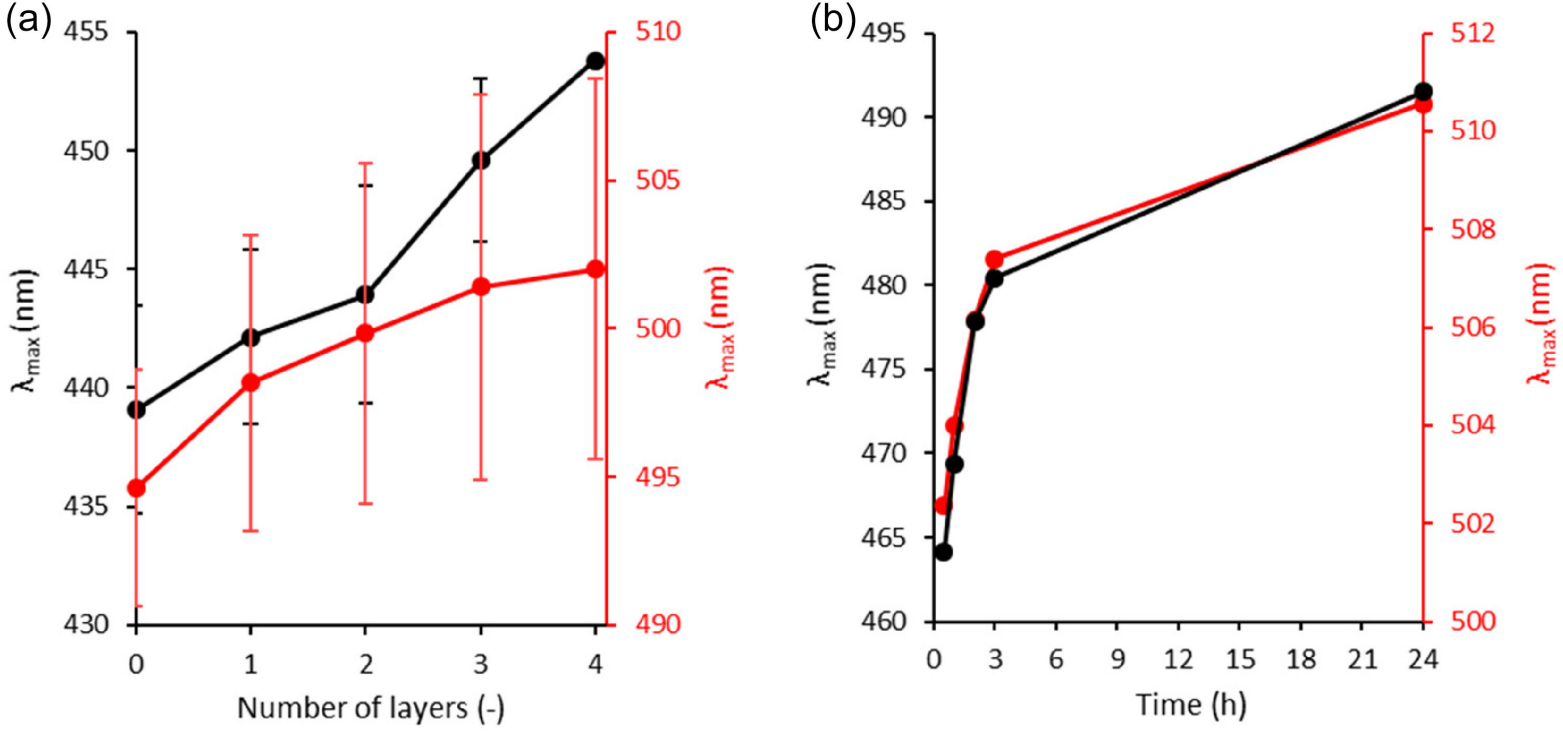
The wavelength of maximal absorbance (*λ*_max_) for the (a) LbL‐ and (b) PANI‐stabilized Au_0.3_Ag_0.7_ (black, left *y*‐axis) and Au_0.7_Ag_0.3_ NP suspensions (red, right axis) resp. as a function of the number of layers or the polymerization time. The error bars in (a), based on the standard deviation, predominantly correspond to the variations in *λ*_max_ for bare NPs and not the variation in redshift with increasing layer number. The latter appeared to be up to ~3 nm.

Based on our previous work, an equimolar mixture of Au_0.__3_Ag_0.__7_ and Au_0.__7_Ag_0.__3_ nanoparticles was selected as a simplified “rainbow” system. Together, these two alloy compositions provide a broad plasmonic absorption band covering a large fraction of the visible solar spectrum (Figure 1a) while greatly simplifying the synthesis compared with the original nine‐component rainbow catalyst (Figure S3) [17, 18]. They were selected for further stabilization with insulating (LbL) and conductive (PANI) polymer shells (Figure 1a–c). In the LbL process, alternating deposition of oppositely charged polyelectrolytes gradually increased the shell thickness, resulting in a progressive redshift of up to ca. 3 nm per layer in *λ*_max_, consistent with the literature (Figure 2a) [18]. The PANI method involved in situ polymerization of aniline at the metal surface, where shell thickness and *λ*_max_ increased over time (Figure 2b). Direct comparison with LbL samples is hard due to different dielectric properties of both polymeric systems, yet PANI samples showed a similar stagnation in redshift after ca. 3 h, coinciding with the solution becoming dark and the appearance of free PANI in the solution. This was confirmed by an additional absorption band at 700–900 nm (Figure S4) [25]. Notably, the PANI shells exhibited larger standard deviations in average thickness than the LbL shells, reflecting the superior thickness control of the latter method. This is consistent with the broader shell size distributions observed for PANI coatings (Figure S5).

**FIGURE 3:**
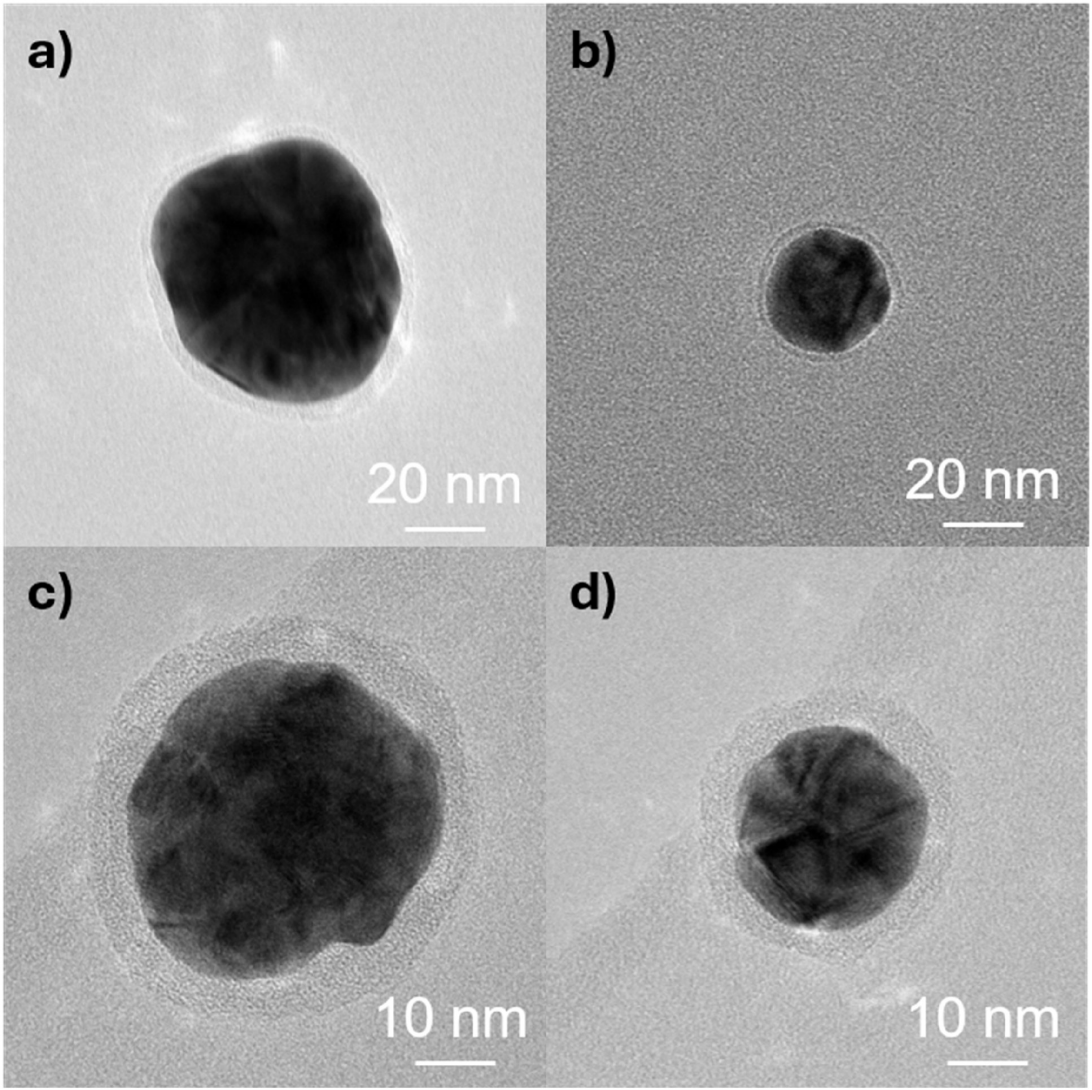
TEM images of (a,b) LbL‐ and (c,d) PANI‐stabilized (a,c) Au_0.3_Ag_0.7_ and (b,`d) Au_0.7_Au_0.3_. A polymer shell is clearly visible surrounding the metal cores in all cases.

BF‐TEM was used to determine the shell thickness. The Au_0.__3_Ag_0.__7_ and Au_0.__7_Ag_0.__3_ cores measured (49 ± 4) nm and (32 ± 5) nm, respectively (Figure 3). After four polymer layers, the LbL shells reached a thickness of (5 ± 1) nm and (2.3 ± 0.5) nm for Au_0.__3_Ag_0.__7_ and Au_0.__7_Ag_0.__3_ NPs, respectively [18]. The PANI shells were of the same order of magnitude: (5 ± 2) nm and (3 ± 1) nm, respectively. Such thin shells are advantageous for retaining NFE at the outer surface without compromising stability, as demonstrated previously [22].

**FIGURE 4:**
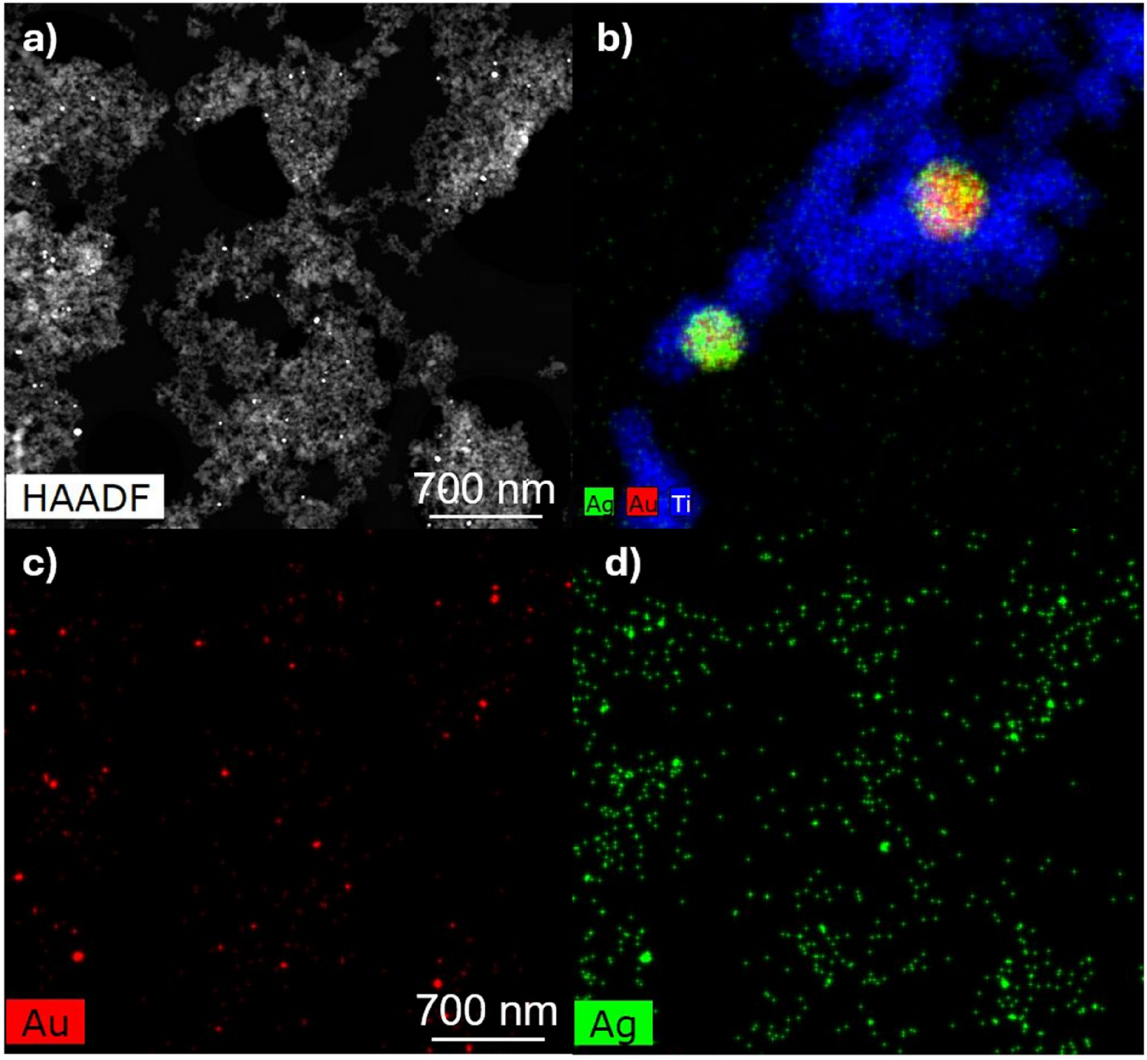
(a) HAADF–STEM image of P25 + 2wt% PANI‐stabilized “rainbow” NPs. (b) EDXS maps of P25 + 2wt% PANI‐stabilized “rainbow” NPs. EDXS map of image (a) for (c) gold and (d) silver.

HAADF–STEM–EDX mapping clearly reveals the presence of discrete bimetallic nanoparticles anchored on the TiO_2_ support (Figure 4a). The nanoparticles exhibit strong high‐Z contrast in HAADF–STEM imaging. EDX analysis reveals co‐localization of Au and Ag signals, consistent with the formation of bimetallic Au–Ag particles (Figure 4b). The particles are typically in the order of a few tens of nanometers and are sparsely distributed over the TiO_2_ surface, resulting in a homogeneous metal dispersion (Figure 4a,c,d).

Successful loading of plasmonic NPs onto TiO_2_ was also confirmed by DRS (Figure 1d), which show distinct plasmon absorption bands in the visible range. The bandgap of the photocatalyst remained nearly unchanged after photo‐impregnation; Kubelka–Munk analysis yielded values similar to pristine TiO_2_ (~3.2 eV) (Table S1). P25 was chosen as the TiO_2_ substrate as it proved to be superior compared to pure anatase and PC500 in earlier comparisons [18]. The bandgap was determined to be 3.15 eV for PANI and 3.19 eV for LbL‐stabilized samples, which is very close to the value for pristine P25, as expected. BET analysis showed similar specific surface areas (~50 m^2^/g^−^^1^) for both bare P25 and P25 decorated with PANI‐ or LbL‐coated nanoparticles (Figure S6) [18]. P25 demonstrates mainly interparticle porosity, and at the very low metal loadings employed in this work, no significant loss of surface area due to pore blocking is expected, which is fully confirmed by the sorption data.

### 3.2 Polymer Stabilization of Plasmonic “Rainbow” Photocatalyst

**FIGURE 5:**
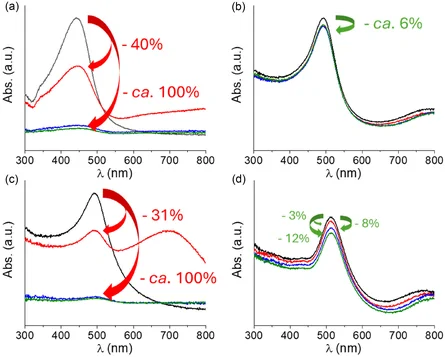
UV–vis absorbance spectra for both non‐stabilized (a,c) and PANI‐stabilized (b,d) Au_0.3_Ag_0.7_ (a,b) and Au_0.7_Ag_0.3_ (c,d) NP suspensions before (black), immediately (red), 24 h (blue), and 42 h (green) after the addition of 150 μL of a 1 M NaCl solution to 1.5 mL NP suspension. The values and arrows in green and red indicate the decrease of the initial maximal absorbance, corrected for dilution and formation of degradation products.

Stability tests based on salt addition and heat treatment confirmed the robustness of the PANI coatings. PANI Au_*x*_Ag_1−*x*_ NPs showed no discoloration even after 2 days, whereas bare Au_*x*_Ag_1−*x*_ NPs discolored immediately upon salt addition (Figure S7). UV–vis quantification revealed minimal losses for PANI‐coated NPs (6%–‍12%), while uncoated NPs lost all optical properties after 42 h, consistent with previous reports (Figure 5) [18]. For comparison, LbL‐stabilized Au_0.__3_Ag_0.__7_ and Au_0.__7_Ag_0.__3_ retained only 52% and 80% stability after 42 h, respectively [18]. The superior stability of PANI‐coated NPs can be attributed to several synergistic effects: (i) Although protonated PANI is not electrostatically repulsive toward chloride ions, its compact morphology and low permeability strongly hinder chloride access to the metal surface while simultaneously suppressing Ag^+^ migration, effectively preventing AgCl formation [26, 27, 28]. (ii) The conformal polymer shell offers strong steric and adhesive stabilization against aggregation and sintering, consistent with the enhanced resistance observed after heat treatment (Figure S8) [29]. In contrast, LbL multilayers are highly hydrated and ion permeable, allowing chloride ions to penetrate the film and reach the metal core [30, 31]. Altogether, these combined effects explain the markedly improved optical and structural stability of PANI‐coated Au_*x*_Ag_1−*x*_ nanoparticles in saline environments.

### 3.3 H_2_ Evolution

#### 3.3.1 Plasmonic “Rainbow”–TiO_2_ in Freshwater

**FIGURE 6:**
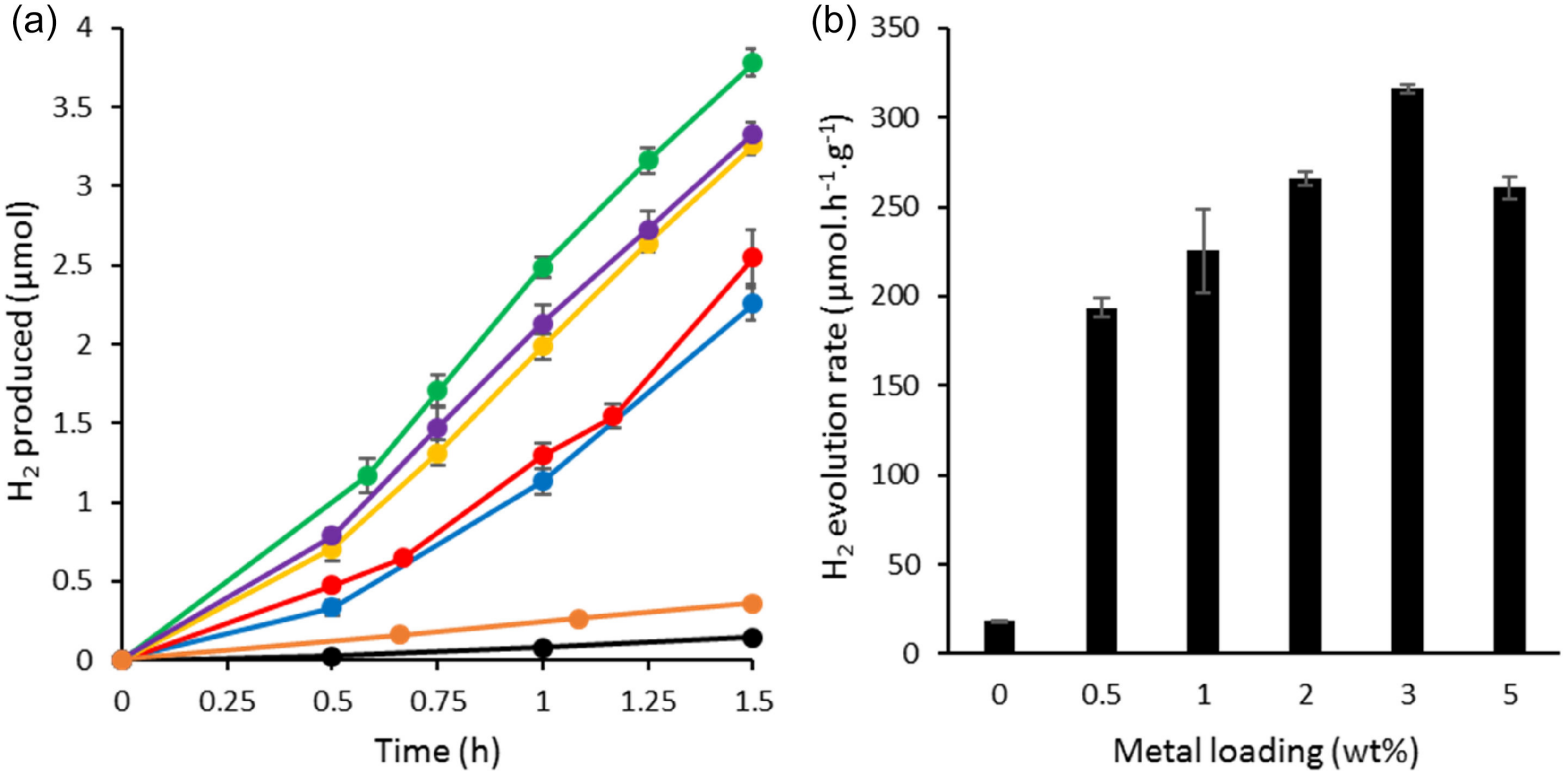
(a) H_2_ evolution curves for P25 + 0 (black), 0.5 (blue), 1 (red), 2 (yellow), 3 (green) and 5 wt% (purple) “rainbow” NPs under 100 mW cm^−2^ AM1.5G simulated sunlight in a pure water/methanol mixture (7:1). The H_2_ evolution curve for 0.2 mg of colloidal Au_*x*_Ag_1*−x*_ NPs alone (orange) is given as well. (b) Summary of the H_2_ evolution rates for different metal loadings. Note that the error bars for (a) are based on the standard deviation of the measurements, while these for (b) are based on the error on the slope of the linear part of the H_2_ evolution curve.

The synthesized plasmonic hybrid samples were all tested for their H_2_ evolution activities. H_2_ was first produced from a pure water/methanol mixture (7:1) in the slurry batch mode. The most appropriate loading of plasmonic “rainbow” NPs was determined by means of 1.5 h measurements using AM1.5G simulated sunlight (100 mW cm^−2^). The H_2_ evolution curves are shown in Figure 6a. In a general measurement, first, there is a lag phase of roughly 30 min, generally attributed to H_2_ transfer from the liquid to gas phase. Afterward, a linear zero‐order pure kinetic regime is reached, which is consistent with the literature [32, 33].

P25 showed only minor H_2_ production ((18.1 ± 0.7) µmol g^−1^ h^−1^), whereas plasmon‐modified samples exhibited a clear enhancement up to a factor of 17 for the best performing composite (Figure 6b). The P25 + 2 wt% composite outperformed the sum of the individual activities of P25 and 0.2 mg of colloidal plasmonic NPs (orange curve in Figure 6a), corresponding to the same NP content as in 10 mg of the hybrid sample. Such poor activity for pristine TiO_2_ is consistent with the literature [34, 35, 36, 37, 38], while notable H_2_ evolution from pure plasmonic particles typically occurs only under high‐intensity illumination (W cm^−2^ range) [12, 39]. The enhancement is consistent with plasmonic mechanisms commonly reported for Au–Ag/TiO_2_ systems, including NFE and direct electron injection (DEI) [12, 13, 14, 15, 40]. The relative contributions of these mechanisms cannot be resolved from the present experiments. At higher metal loadings, the hydrogen evolution rate reaches a broad optimum and slightly decreases thereafter (Figure 6b). Such behavior is commonly reported for metal‐modified TiO_2_ photocatalysts and is generally attributed to increased light shielding, partial coverage of photocatalytically active TiO_2_ surface sites, and enhanced charge recombination at excessive cocatalyst loadings [41, 42, 43]. The 3 wt% sample showed the highest activity ((316 ± 3) µmol g^−1^ h^−1^), in good agreement with reported plasmonic TiO_2_ systems [44]. Xia et al. reported enhancement factors from 1.7 to 223 (average ≈ 38) for modified TiO_2_ [45], which is well in line with our observations. Similar activities were also reported for Au‐ and Ag‐modified TiO_2_ [35, 45, 46]. Introducing Pt further increased H_2_ evolution by an order of magnitude, reflecting its superior electron sink capacity and low hydrogen overvoltage (HOV) [47]. Cheng et al. observed a 60‐fold enhancement for PtAu/TiO_2_ compared with Au/TiO_2_ (8010 vs. 134 µmol g^−1^ h^−1^) under Xe illumination [35], while Bahnemann et al. found Pt/TiO_2_ outperforming Ag and Au analogues (ca. 1 mmol g^−1^ h^−1^ vs. 77 and 307 µmol g^−1^ h^−1^, respectively) [46]. As Tanaka et al. reported, H_2_ evolution correlates inversely with HOV (Pt > Pd > Ru > Rh > Au > Ag > Cu > Ir) [48].

#### 3.3.2 Stabilized Metal@polymer Core–Shell “Rainbow”–TiO_2_ in (Sea)water

**FIGURE 7:**
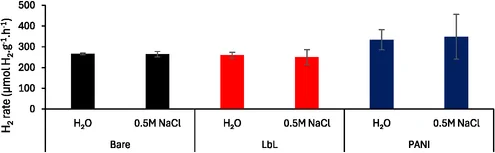
Summary of the H_2_ evolution rate for P25 + 2 wt% bare “rainbow” NPs (black), LbL‐ (red), and PANI (blue)‐stabilized “rainbow” NPs.

Figure 7 presents the H_2_ evolution activities in pure water and simulated seawater under simulated sunlight. Although simulated seawater does not mirror the full ionic complexity of natural seawater, NaCl‐based simulated seawater is widely used as a first model electrolyte in seawater‐splitting studies [49, 50, 51, 52]. Chloride alone already causes severe instability of unprotected nanoparticles, while the polymer‐stabilized systems retain their optical and photocatalytic performance after prolonged NaCl exposure, as discussed earlier (Figure S7). Future work could extend these tests to more complex artificial and natural seawater to evaluate additional effects from Mg^2+^, Ca^2+^, SO_4_^2^^−^ and HCO_3_^−^ such as scaling, passivation, and competitive adsorption.

**FIGURE 8:**
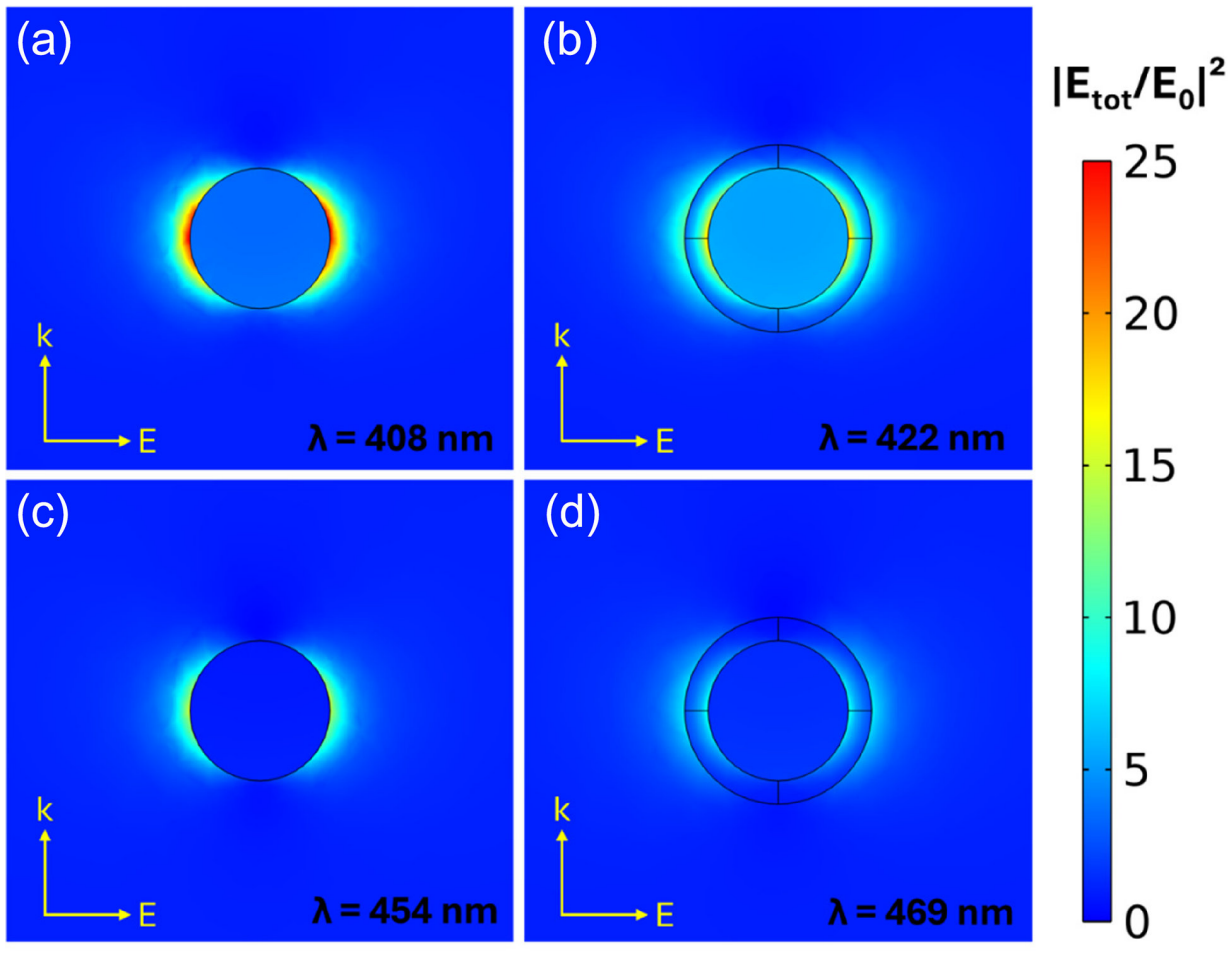
Near‐field intensity enhancement maps for 15‐nm‐sized bare (a,c) and PANI‐stabilized (b,d) Au_0.3_Ag_0.7_NPs (a,b) and Au_0.7_Ag_0.3_ NPs (c,d), plotting the normalized ratio of the squared total electrical near field and the squared incident electrical field (|*E*_tot_/*E*_0_|^2^) for the wavelength (*λ*) of maximal NFE. The PANI shells have a thickness of 5 nm. Note that the enhancement of TiO_2_’s activity by near field occurs proportionally to |*E*_tot_/*E*_0_|^2^. The wave direction (*k*) and energy (*E*) are given as well.

To enable a direct comparison with LbL‐stabilized samples from previous work, a 2 wt% NP loading on P25 was selected [18]. In pure water, the LbL‐stabilized catalysts showed a slightly lower activity than the bare plasmonic sample ((259 ± 14) vs. (266 ± 4) µmol H_2_ g^−1^ h^−1^), which can be rationalized by the insulating nature of the shell that partially hinders hot electron transfer. Nevertheless, the coating effectively protects the nanoparticles from oxidation during synthesis. In contrast, the PANI‐stabilized catalysts exhibited the highest activity ((334 ± 48) µmol H_2_ g^−1^ h^−1^), corresponding to a 29% increase relative to the LbL sample and a 26% enhancement compared to the pristine composite. The larger error bar reflects the high activity approaching the detector’s upper limit. While COMSOL simulations indicate a modest reduction in the maximum near‐field intensity upon the introduction of the PANI shell (Figure 8), the observed activity enhancement suggests that near‐field effects cannot solely account for the observed trends. Instead, the conductive PANI shell likely also facilitates electron transfer from the plasmonic core into the semiconductor. To assess whether the conductive polymer shell remains electronically accessible, an XTT superoxide formation assay was performed (Figure S9). The PANI‍‐‍coated “rainbow” NPs on SiO_2_ retained approximately 60% of the XTT conversion efficiency of the corresponding uncoated “rainbow” nanoparticles after 1 h of irradiation, demonstrating that electron transfer‐dependent oxygen reduction remains possible despite the presence of the polymer shell. As a qualitative comparison with an insulating encapsulation strategy, XTT measurements were also performed on Au_0.__3_Ag_0.__7_ nanoparticles coated with four polyelectrolyte layers, showing almost complete suppression of the electron transfer reaction. Though the XTT assay does not directly probe charge injection kinetics at the Au–Ag/PANI/TiO_2_ interface, the results indicate that electron transfer remains enabled through the PANI shell. In combination with the COMSOL simulations, this suggests that preserved charge transfer may contribute to the enhanced photocatalytic hydrogen evolution, while the overall activity trends remain consistent with NFE being the dominant plasmonic mechanism, in agreement with previous work on conductive and insulating spacer layers [40].

Notably, the simulations show that the strongest electromagnetic enhancement is predominantly localized within the PANI shell itself. However, as this region is not in direct contact with the semiconductor, it is unlikely it serves as an active site for near‐field‐driven charge generation. Consequently, we hypothesize the PANI coating to promote charge transfer pathways that are more critical for photocatalytic performance while simultaneously ensuring chemical stability.

In simulated seawater, all samples show comparable H_2_ evolution activities to pure water. The initial stability of the bare plasmonic NPs, despite the absence of a protective shell, indicates successful and good immobilization on the P25 support, preventing direct aggregation as seen in the salt addition tests. The oxygen‐free atmosphere during photocatalysis further minimizes oxidation losses and catalyst degradation. Moreover, no adverse effects related to the saline medium, such as Cl_2_ evolution or ion adsorption, were observed. IC of the particle‐free solutions before and after the reaction confirmed unchanged ion concentrations (Figure S10), indicating negligible chloride‐based adsorption or reaction.

**FIGURE 9:**
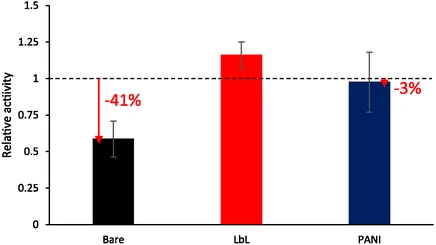
Relative activity for new samples and after 1 month of aging for P25 + 2 wt% “rainbow” (black), 2 wt% LbL‐ (red) and PANI (blue)‐stabilized “rainbow”.

It should be stressed that the results shown thus far are obtained using fresh samples tested shortly after synthesis. In that sense, it is not so surprising that the observed performances in a first seawater experiment are similar. Hence, to adequately assess long‐term stability, the same samples were retested after 1 month of storage in a saline environment in the dark (Figure 9). While the activity of the bare P25 + 2 wt% “rainbow” sample decreased by 41%, the H_2_ evolution rates of both PANI‐ and LbL‐stabilized “rainbow” catalysts remained unchanged. The slight increase in activity observed for the LbL‐coated sample remains within the experimental error. In parallel, stability under photocatalytic operating conditions was evaluated through four consecutive 4‐h‐long illumination cycles, corresponding to 16 h of cumulative illumination (Figure S11). All catalysts retained at least ca. 70% of their initial activity, with relatively small differences in activity retention between the bare, LbL‐, and PANI‐stabilized catalysts. Both stability experiments provide complementary information for operational and dark storage conditions. While substantial and similar activity is retained during prolonged illumination of all samples, the 1‐month storage experiment reveals a pronounced difference between the bare and stabilized catalysts. This demonstrates the importance of stabilizing the metal NPs to preserve catalyst activity during prolonged exposure to a saline environment, including nonoperational periods such as nighttime or dark storage. These findings also underscore an important consideration when evaluating activity data in the literature, which are commonly reported for materials tested shortly after synthesis. Our results show that combining operational stability testing with retesting after a prolonged period of aging provides valuable information on catalyst stability that may otherwise remain overlooked.

#### 3.3.3 Cost‐Effectiveness Analysis of Plasmonic Photocatalysts for H_2_ Evolution

Given the scarcity and cost of Pt and Pd, performance evaluation through cost‐effectiveness metrics, such as the average cost‐effectiveness ratio (ACER, Equation (1)) and incremental cost‐effectiveness ratio (ICER, Equation (2)), is introduced [53].



(1)

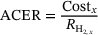

(2) $$\text{ICER}=\frac{{\text{Cost}}_{x}-{\text{Cost}}_{{\mathrm{TiO}}_{2}}}{R_{H_{2\text{,}x}}-R_{H_{2,{\mathrm{TiO}}_{2}}}}$$

**FIGURE 10:**
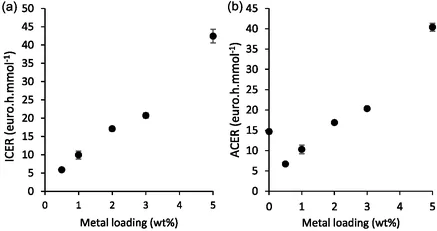
(a) Incremental and (b) ACER (in euro h nmol^−1^ H_2_) as a function of the plasmonic metal loading.

With Cost_*x*_, ${\mathrm{Cost}}_{{\mathrm{TiO}}_{2}}$, $R_{H_{2}{,}_{x}}$_*,*_ and $R_{H_{2},{\mathrm{TiO}}_{2}}$ the cost and H_2_ production rate of sample *X* or pristine TiO_2_, respectively. ACER expresses the average additional cost required to achieve a given increase in hydrogen yield, whereas ICER quantifies the cost of each incremental performance gain upon increasing the plasmonic metal loading. While higher plasmonic loadings lead to slightly increased hydrogen evolution rates, the ICER rises sharply, indicating diminishing economic returns. The most cost‐effective catalyst composition is therefore identified by a minimum in ACER, accompanied by a pronounced increase in ICER when further increasing the metal loading. From Figure 10a,b, P25 + 0.5 wt% “rainbow” NPs are the most cost‐effective composition for photocatalytic H_2_ evolution under simulated sunlight, given the minimum in ACER. While higher loadings (2–3 wt%) show slightly greater activities, the gain does not justify the increased cost associated with larger quantities of metal precursors (increasing ICER), thus shifting the economic optimum toward lower loadings.

For comparison with the literature, the ACER was estimated from precursor costs, focusing on the main metal reagents (Table S2). Table 1 summarizes photocatalytic hydrogen evolution systems reported in the literature together with their reaction conditions, hydrogen evolution rates, and corresponding calculated ACER values. Though direct quantitative comparison of catalytic performance is often complicated by differences in used light sources, intensities, sacrificial agents, and reactor configurations, ACER provides a useful first‐order metric to assess the balance between photocatalytic performance and material cost.

**TABLE 1:** H_2_ evolution studies using (mostly) photocatalytic slurries, given with their reaction conditions, activity, and ACER.

Photocatalyst	Light (intensity)	Reaction medium (temperature, pressure)^a^	Activity, mmol g^−1^ h^−1^	ACER,^b^ euro h mmol^−1^	References
Au@Cu_2_FeSnS_4_	Solar (100 mW cm^−2^)	0.25 M Na_2_SO_3_ and 0.35 M Na_2_S in H_2_O	0.090	641^c^	[54]
0.5 wt% AuPd/g‐C_3_N_4_	>400 nm (35 mW cm^−2^)	10 vol.% triethanolamine in H_2_O	0.326	4.81	[55]
1 wt% Pt/ Brookite TiO_2_	UV–vis (950 mW cm^−2^)	H_2_O without scavengers	1.2	9.45	[56]
Pt/TiO_2_	UV–vis (>320 nm, 558 mW cm^−2^)	1.09 M glycerol (pH 7.7)	3.09	0.81	[57]
TiO_2_NTs‐Au@Pt	Solar (100 mW cm^−2^)	0.5 M NaSO_4_ in methanol/H_2_O (1:4 v/v); Ar	2.971	45.7^c^	[47]
AuPt/Ti^3+^ inverse opal TiO_2_	UV–vis (100 mW cm^−2^ for 350–700 nm)	10% ethanol in H_2_O (35 °C)	181.77	0.055	[11]
Au@mesoporous TiO_2_ hollow nanospheres	>420 nm (200 mW cm^−2^)	0.1 M ascorbic acid in methanol/H_2_O (1:1 v/v)); N_2_	0.04025	140	[58]
P25 + 0.5 wt% Rainbow NPs	Solar (100 mW cm^−2^)	Methanol/H_2_O (1:7 v/v), N_2_ (21 °C)	0.194	5.42	This work

a If known.

b If the source of the precursor was not specified, the Sigma‐Aldrich product was selected.

c Cost of resp. S and Ti precursor is neglected, since it could not be retrieved and is insignificant compared to the metal precursor cost.

Strikingly, Pt‐based photocatalysts generally exhibit very low ACER values, reflecting their exceptionally high hydrogen evolution rates, which compensate for the high cost of the noble metal cocatalyst. In contrast, many plasmonic systems that do not rely on Pt display substantially higher ACER values, as their lower intrinsic activities are insufficient to offset the cost of Pt.

The plasmonic “rainbow”–TiO_2_ catalyst developed in this work occupies a competitive position among alternative noble metal‐containing photocatalysts, achieving a comparatively low ACER while operating under simulated solar light. This favorable balance originates from the deliberate combination of inexpensive Ag, providing a strong near‐UV plasmonic response, with small amounts of Au, extending absorption toward ~520 nm. Such spectral tuning enhances NFE and DEI while minimizing noble metal usage. Despite operating at lower light intensity, AuPd‐modified g‐C_3_N_4_ only slightly outperformed plasmonic “rainbow”–TiO_2_, likely due to its smaller bandgap (2–3 eV, ~460 nm absorption edge) [55, 59]. As a final comment, it should be highlighted that this cost‐based comparison obviously overlooks material criticality and risk of supply, the latter being region specific.

## 4 Conclusion

This study demonstrates the potential of plasmon‐modified TiO_2_ as a cost‐effective photocatalyst for solar hydrogen generation from seawater. The optimal composition, P25 + 3 wt% “rainbow” Au–Ag NPs, achieved a maximum H_2_ evolution rate of (316 ± 3) µmol g^−1^ h^−1^ in freshwater, while the 0.5 wt% sample offered the best balance between performance and material cost ((194 ± 5) µmol g^−1^ h^−1^). To address the instability of plasmonic metals in saline media, the nanoparticles were encapsulated using two complementary approaches: insulating LbL coatings and conductive polyaniline (PANI) shells. The PANI‐stabilized photocatalysts outperformed both the LbL‐coated and bare samples. Upon exposure to simulated seawater, the bare samples exhibited a 41% activity loss after 1 month, whereas both polymer‐stabilized systems retained their initial performance, positioning both systems at the state of the art compared to existing plasmonic hybrids. Overall, these results establish conductive polymer encapsulation as an effective route to stabilize plasmonic photocatalysts in corrosive environments and represent a significant step toward scalable, stable, and economically viable seawater‐to‐hydrogen conversion.

## Funding

This work was supported by Fonds Wetenschappelijk Onderzoek (Aspirant PhD fellowship 1129726N and Aspirant PhD fellowship 1S28925N) and HORIZON EUROPE Framework Programme (10113111 (Delight)).

## Conflicts of Interest

The authors declare no conflicts of interest.

## Supporting information

Supplementary Material

## Data Availability

The data that support the findings of this study are available from the corresponding author upon reasonable request.
