# Supplementary material for: Hydrogen From Simulated Seawater Using Long‐Term Stable and Cost‐Effective Plasmonic Photocatalysts
Source: ChemSusChem. 2026 Sep 22;19(18):e71072. doi: 10.1002/cssc.71072 (PMC13598271; doi:10.1002/cssc.71072)
Supplement: Supplementary file 1 [file CSSC-19-e71072-s001.pdf]

Supporting information

Hydrogen from Simulated Seawater Using Long-Term Stable and Cost-Effective Plasmonic Photocatalysts

Fons Dingenen,[a,b,*] Manu Donders,[a,b,*] Rajeshreddy Ninakanti,[a,b,c] Christine Vantomme,[a,b] Daniel Arenas-Esteban,[b,c] Rituraj Borah,[a,b] Sara Bals,[b,c] Sammy W. Verbruggen[a,b,†]

[a] Antwerp engineering, photoelectrochemistry and sensing (A-PECS), Department of Bioscience Engineering, University of Antwerp, Groenenborgerlaan 171, 2020 Antwerp, Belgium

[b] NANOlight Center of Excellence, University of Antwerp, Groenenborgerlaan 171, 2020 Antwerp, Belgium

[c] Electron Microscopy for Material Science (EMAT), Department of Physics, University of Antwerp, Groenenborgerlaan 171, 2020 Antwerp, Belgium

* Contributed equally

† Corresponding author: Sammy.Verbruggen@uantwerpen.be

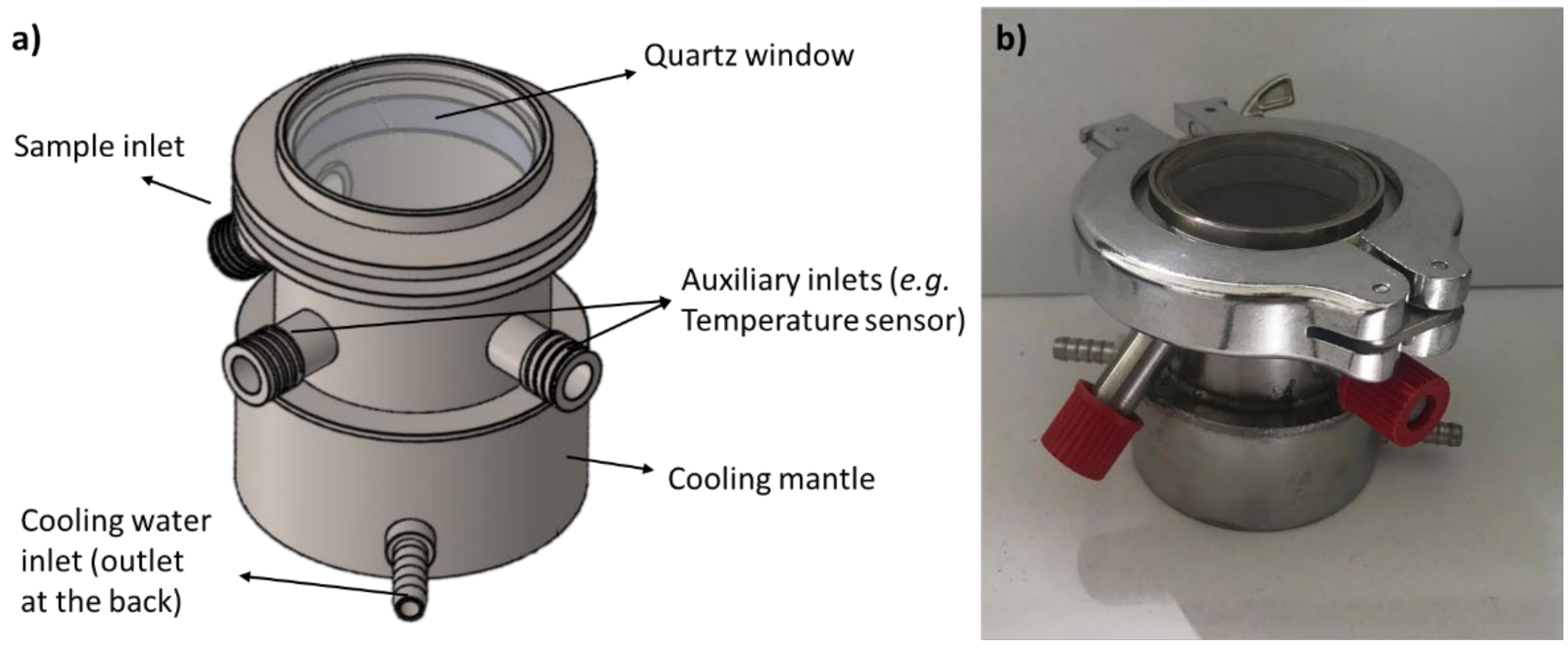

Figure S1: a) Schematic representation and b) image of custom-made batch reactor for photocatalytic hydrogen production from (sea)water.

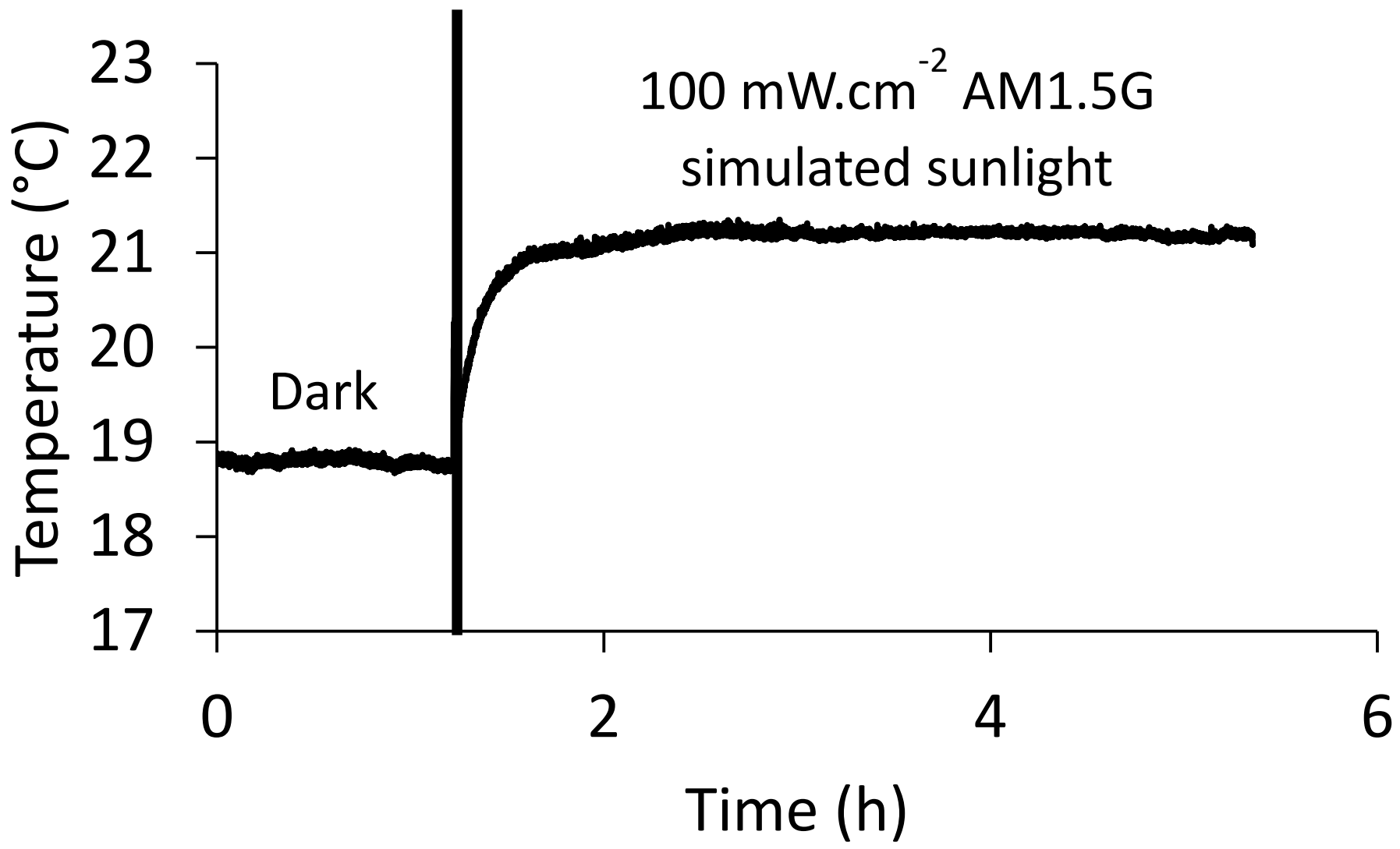

Figure S2: Temperature profile in function of time, showing a 2°C increase caused by the 100 $mW.cm^{-2}$ AM1.5G simulated solar light irradiance.

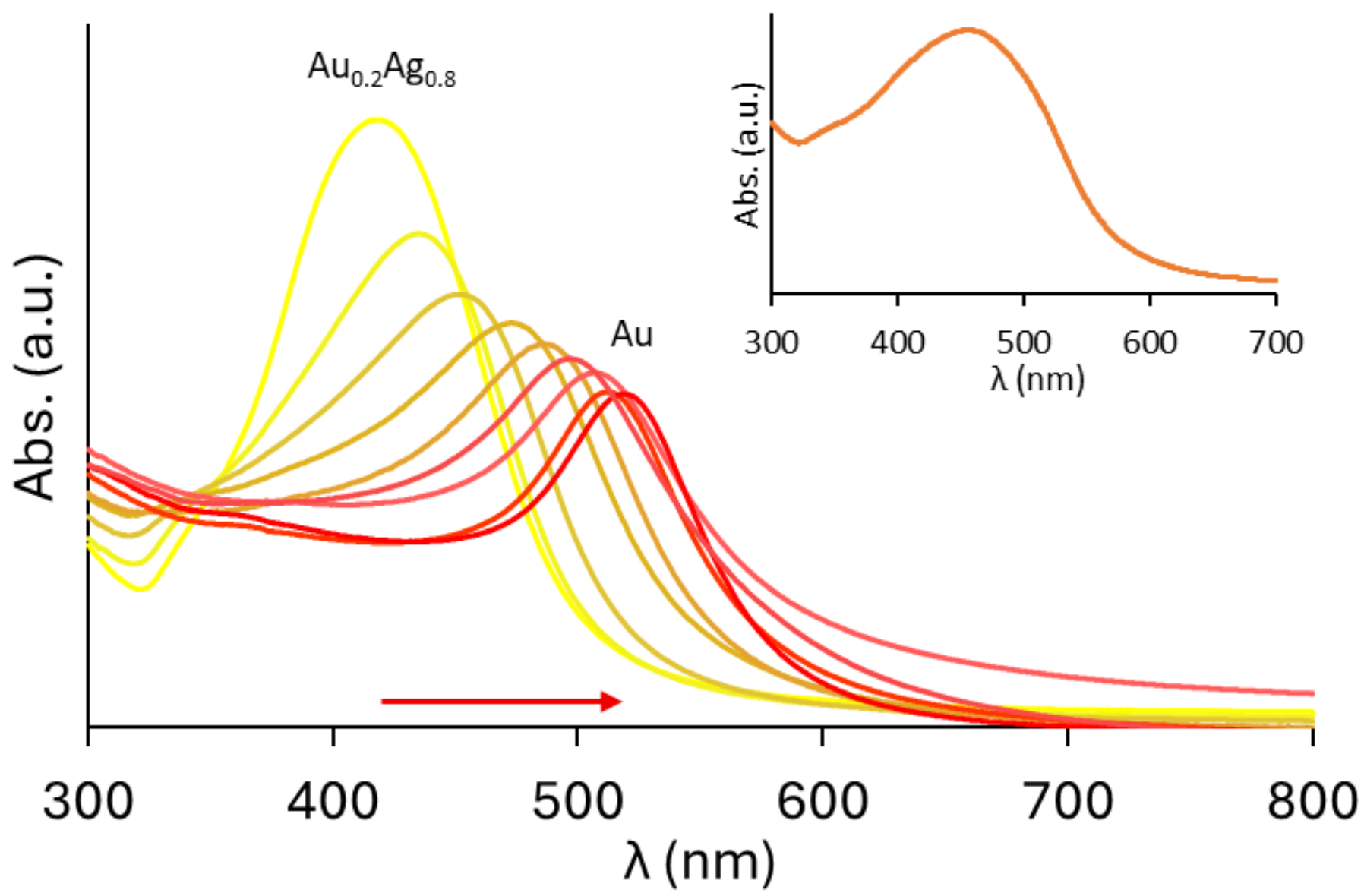

Figure S3: UV-VIS absorption spectra for various Au_xAg_{1-x} suspensions (x ranging from 0.2 to 1 with incremental steps of 0.1), displaying absorption (Abs.) in function of the wavelength (λ). The red arrow indicates the redshift for increasing gold content. Inset: the combined UV-VIS absorption spectrum of the 'rainbow' mixture. As reported by Dingenen *et al*. (2021).[1]

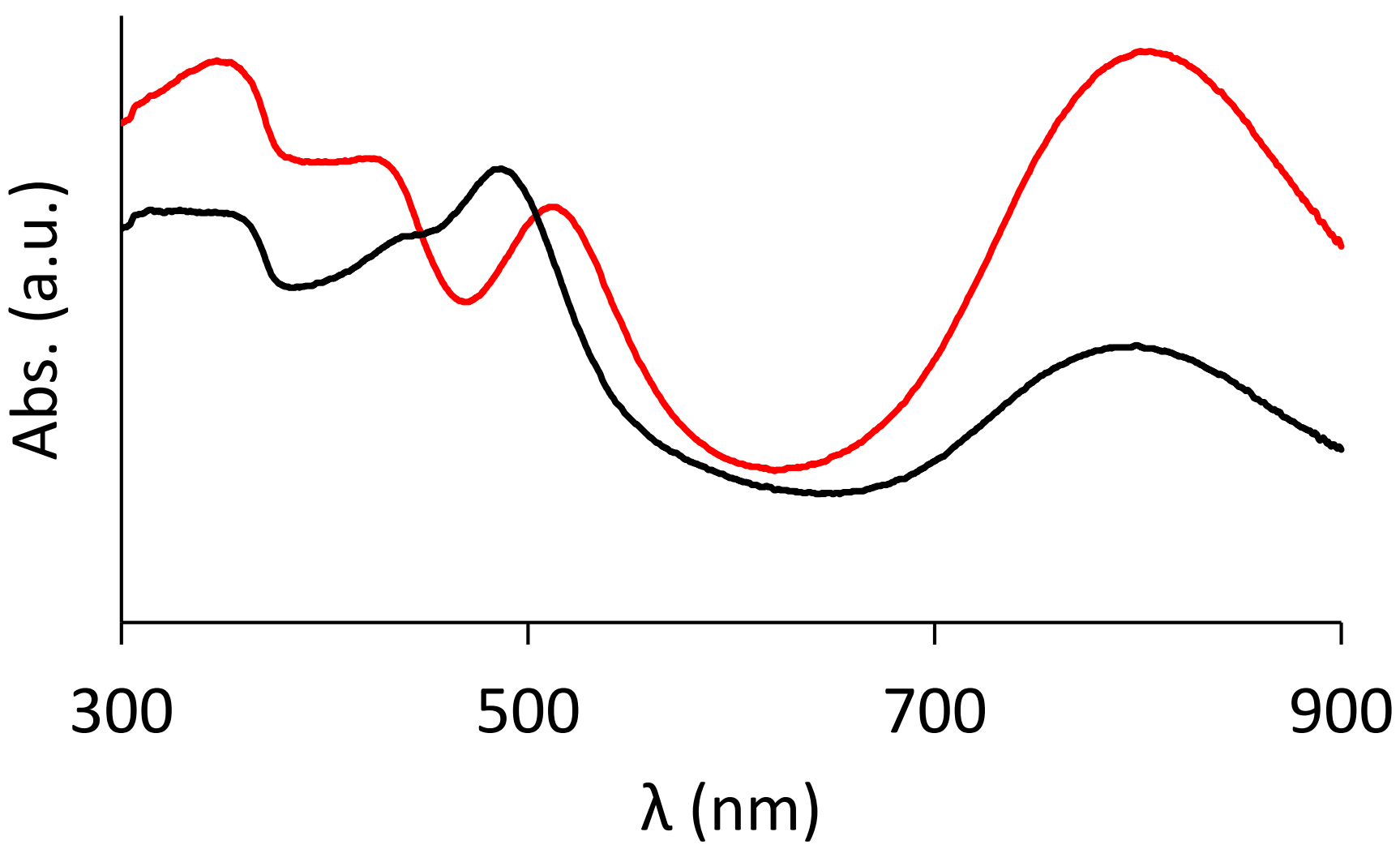

Figure S4: UV-VIS absorption spectra, plotting the absorbance (Abs.) vs. the wavelength of the incident light (λ), for $Au_{0.3}Ag_{0.7}$ (black) and $Au_{0.7}Ag_{0.3}$ (red) after *in-situ* PANI polymerization overnight. The peaks around 350 nm, 430 nm and 810 nm indicate the presence of free protonated polyaniline.[2]

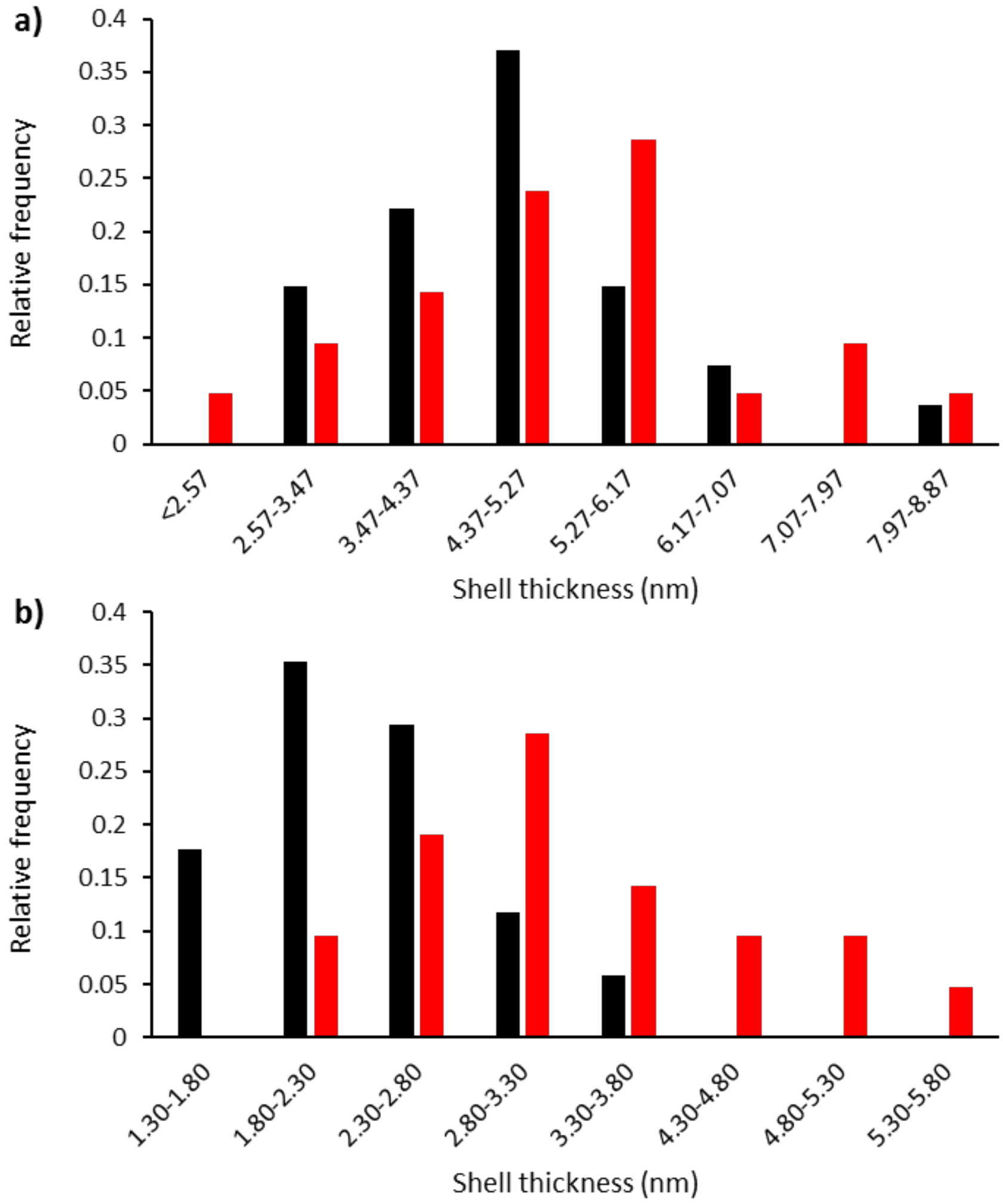

Figure S5: Shell thickness distributions around a) $Au_{0.3}Ag_{0.7}$ and b) $Au_{0.7}Ag_{0.3}$ NPs for both LbL (black) and PANI (red) shells.

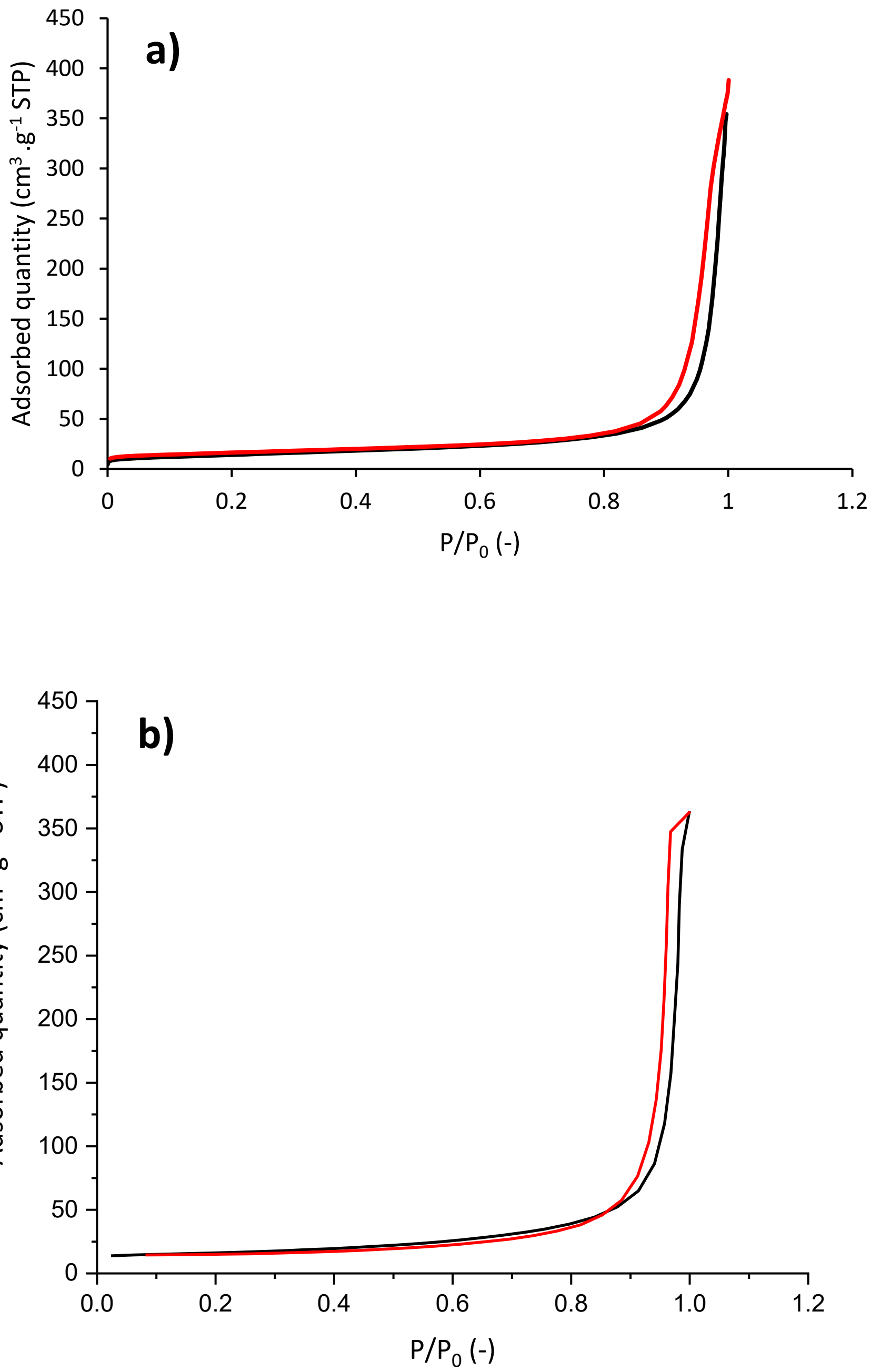

Figure S6: N_2 ad- (black) and desorption (red) curve, plotting the adsorbed quantity (in cm^3.g at standard temperature and pressure (STP)) vs. the pressure ratio (P/P_0), for a representative a) P25 and b) PANI 'rainbow' on P25 sample.

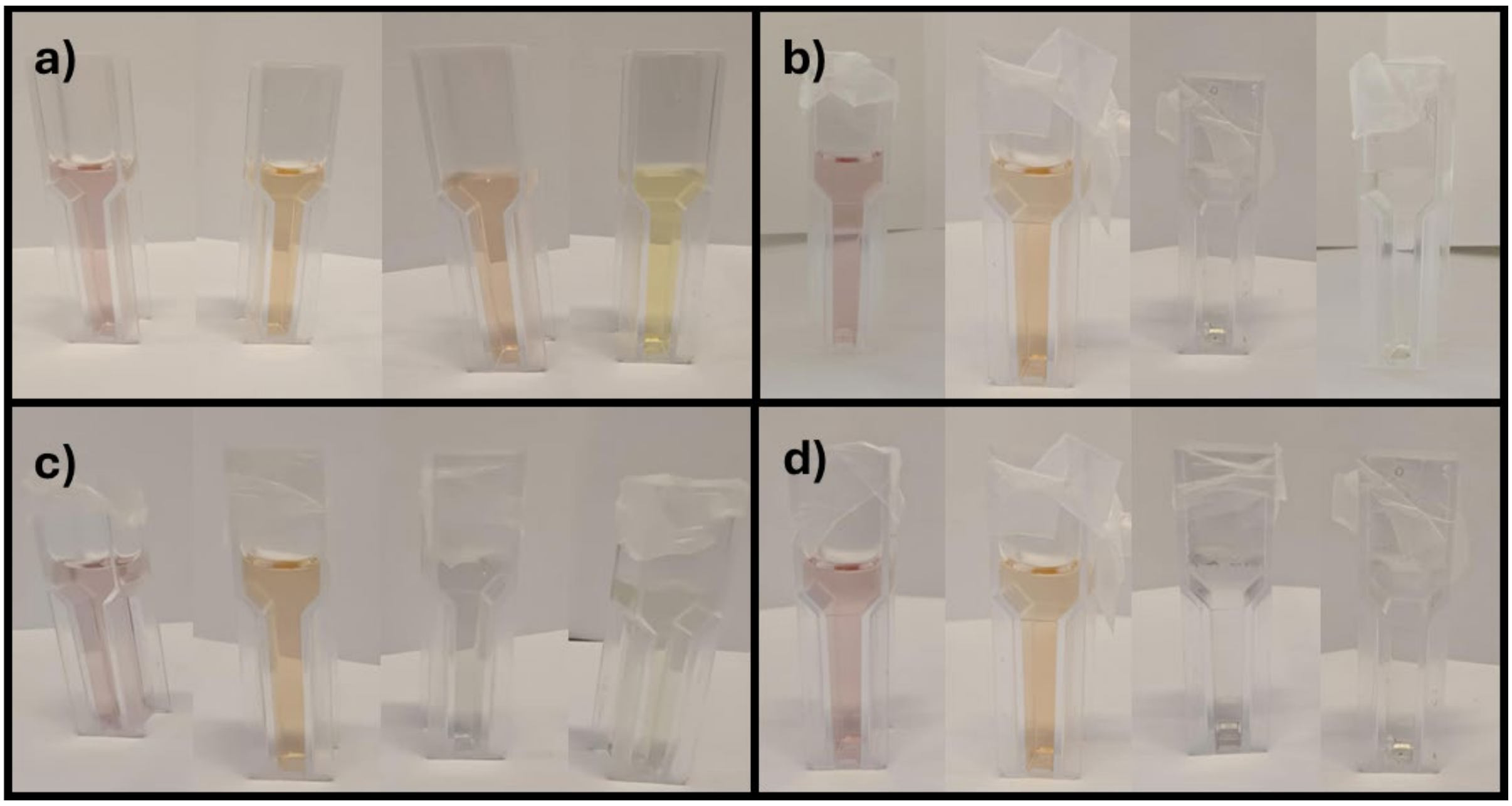

Figure S7: Images of bare and PANI-coated Au_xAg_{1-x} NPs before salt addition (a), immediately after salet addition (b), after 24 hours (c), and after 42 hours (d). Left to right: PANI $Au_{0.7}Ag_{0.3}$, PANI $Au_{0.3}Ag_{0.7}$, $Au_{0.7}Ag_{0.3}$, and $Au_{0.3}Ag_{0.7}$.

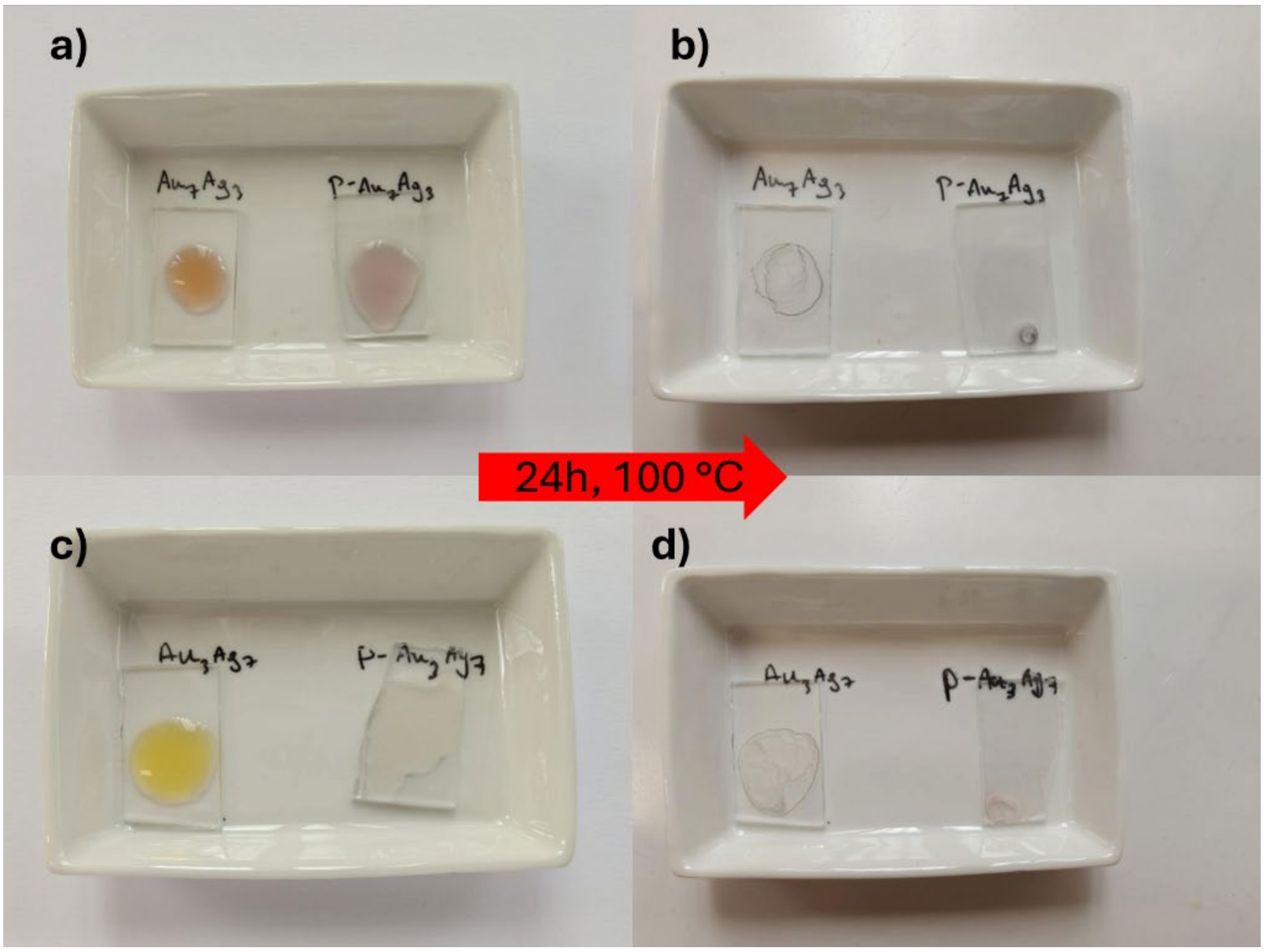

Figure S8: Images of concentrated bare and PANI-coated $Au_{0.7}Ag_{0.3}$ (a,b) and $Au_{0.3}Ag_{0.7}$ (c,d) NPs drops on pre-treated glass slides before (a,c) and after (b,d) heat treatment.

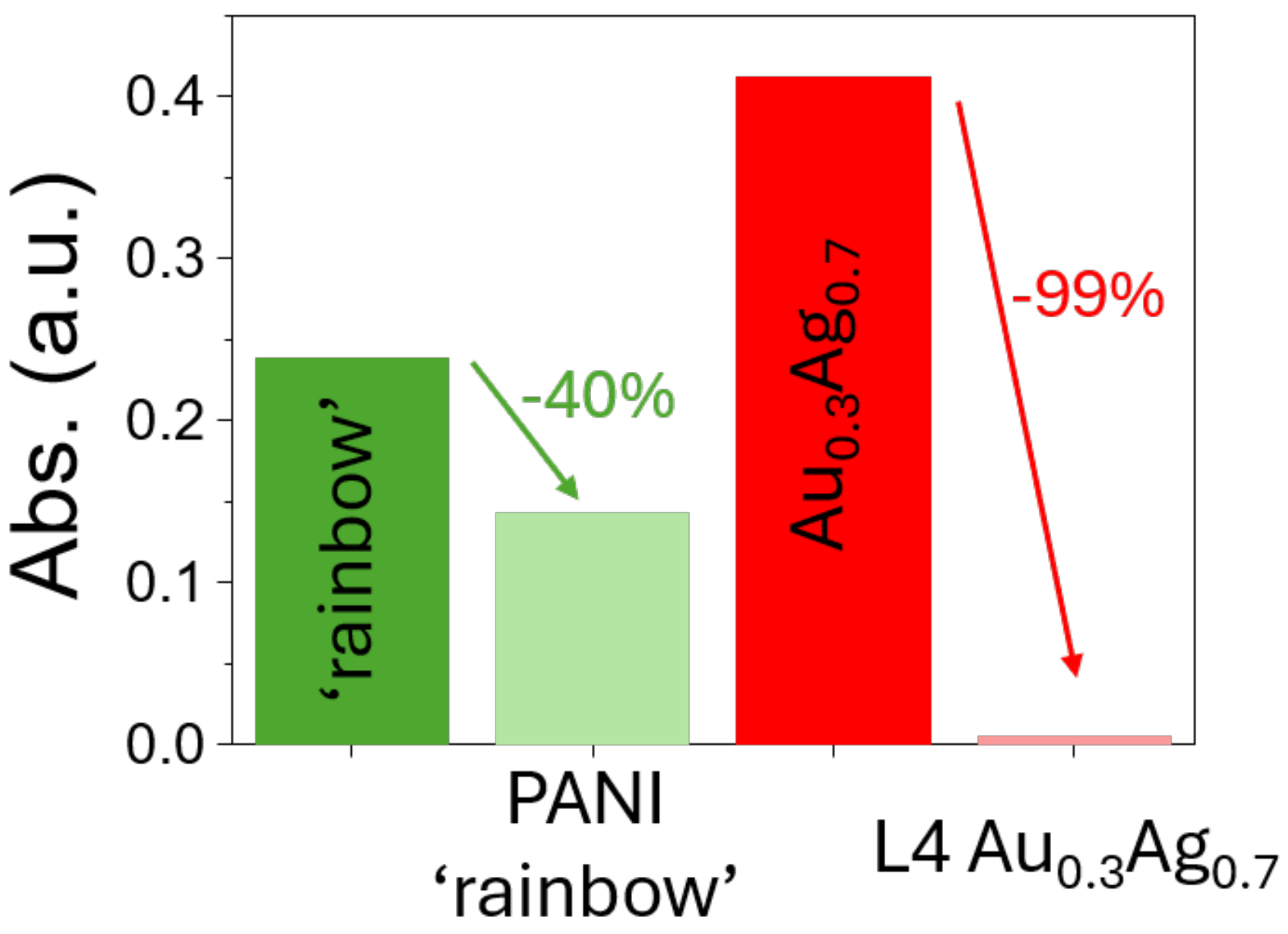

Figure S9: XTT assay after 60 min of AM1.5G irradiation for bare and PANI-coated 'rainbow' nanoparticles and bare and LbL-coated $Au_{0.3}Ag_{0.7}$ nanoparticles immobilized on SiO2. The LbL approach thus uses only the $Au_{0.3}Ag_{0.7}$ component with four polyelectrolyte layers (L4) and represents a qualitative comparison.[1]

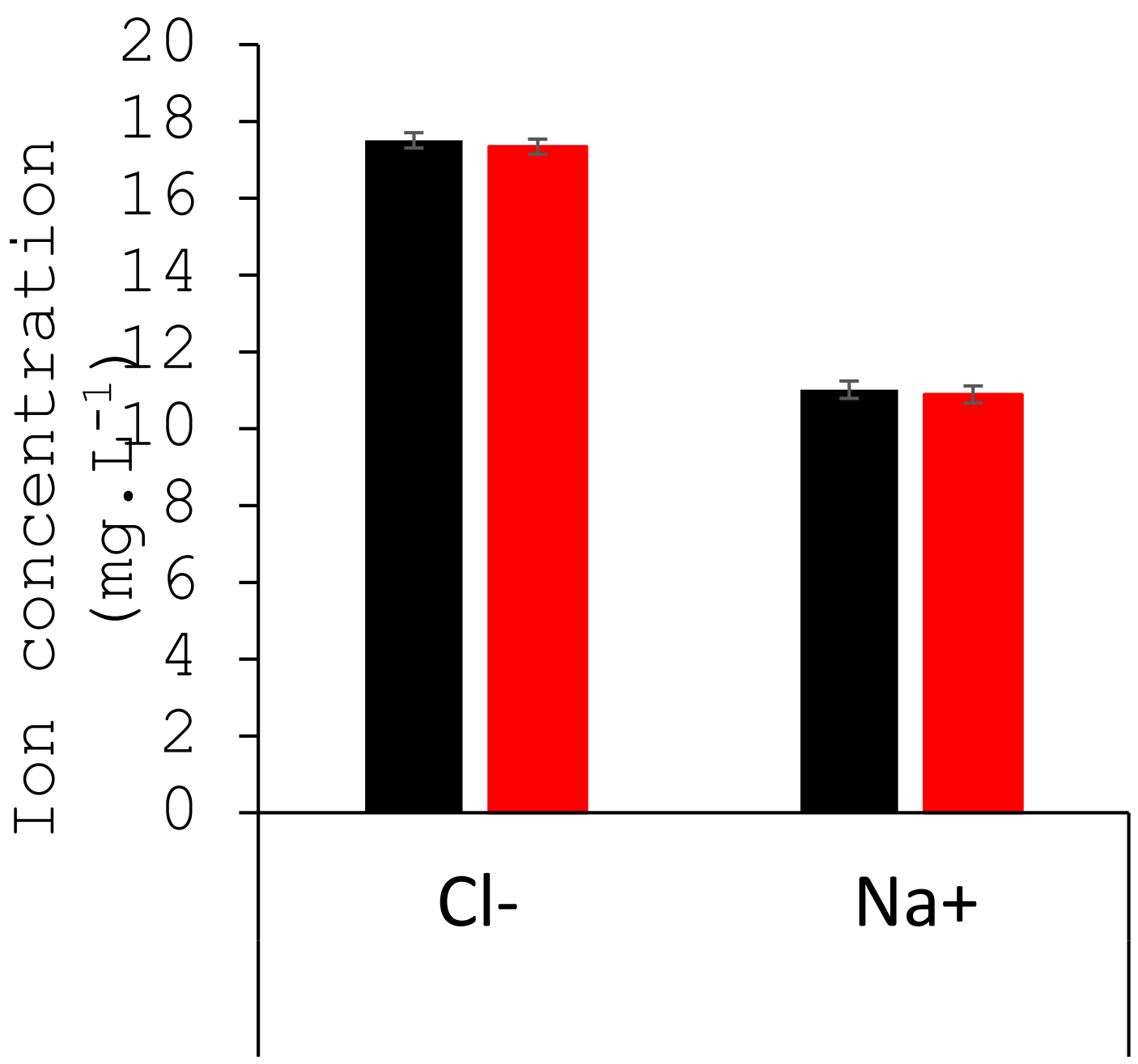

Figure S10: Ion concentration of 0.5 M NaCl before (black) and after (red) H_2 evolution reaction.

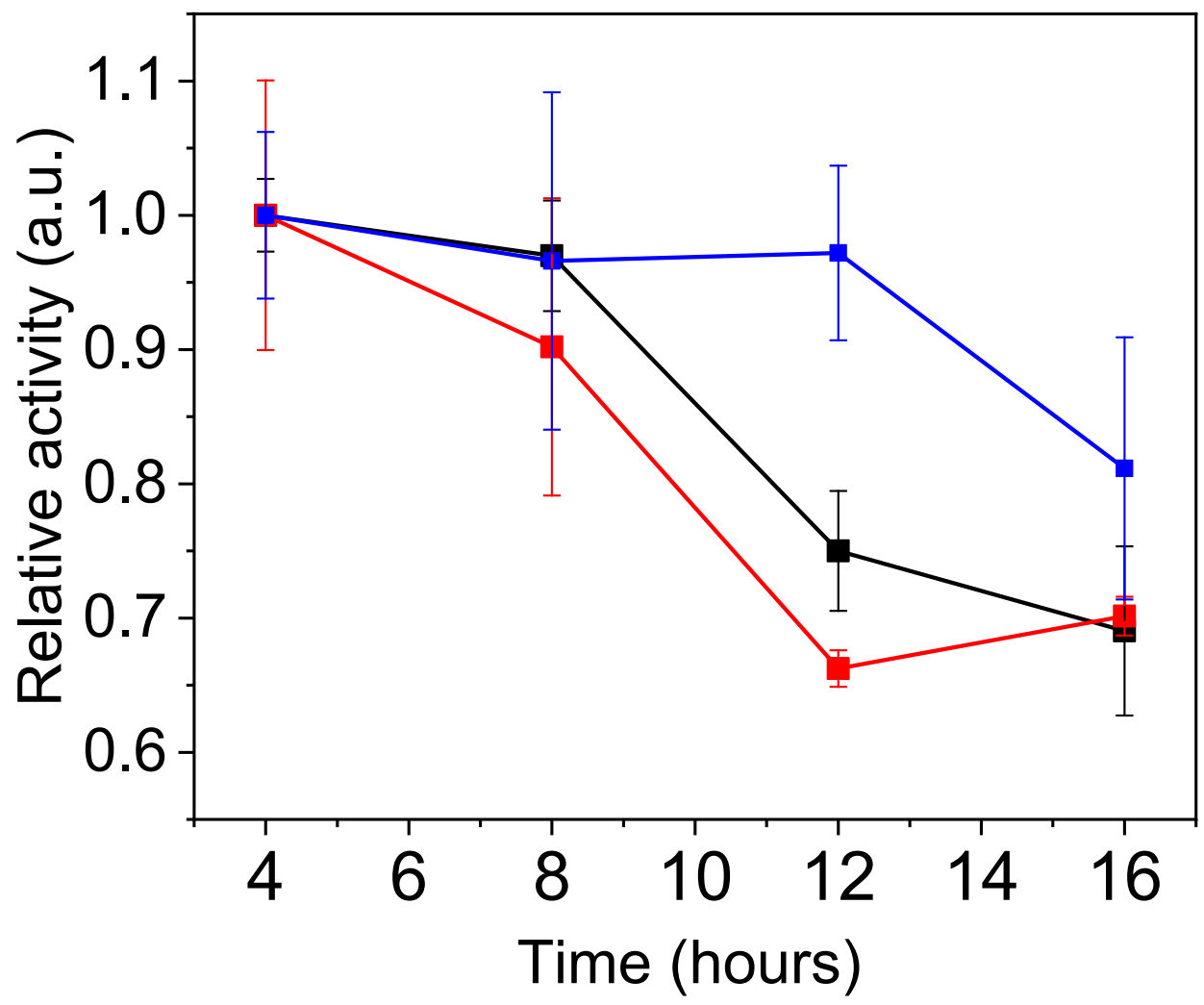

Figure S11: Photocatalytic cycling stability of bare (black), LbL-stabilized (red), and PANI-stabilized (blue) P25 + 2 wt% 'rainbow' catalysts during four consecutive 4 h illumination cycles (16 h total) in simulated seawater.

Tables

Table S1: Summary of band gaps (E_g) for different tested photocatalysts.[1]

Metal loading	Eg (eV)
**0 wt% R**	3.27
**0.5 wt% R**	3.07
**1 wt% R**	3.18
**2 wt% R**	3.18
**3 wt% R**	3.26
**5 wt% R**	3.15
**2 wt% PANI $Au_{0.3}Ag_{0.7}$ + $Au_{0.7}Ag_{0.3}$**	3.15
**2 wt% LbL $Au_{0.3}Ag_{0.7}$ + $Au_{0.7}Ag_{0.3}$**	3.28

Table S2: Summary of the precursor costs, given with their respective producers and purity for largest packaging. As determined in 07/2023.

Precursor	Producer	Purity	Cost (euro.g^{-1} precursor)	Ref.
**$AgNO_3$**	Sigma-Aldrich	ACS reagent, 99.0%	3.26	[3]
**$HAuCl_4$ .3 H_2O**	Sigma-Aldrich	trace metal basis, 99.9%	114	[4]
**H_2PtCl_6 .6 H_2O**	Sigma-Aldrich	ACS reagent, 37.50% Pt basis	414	[5]
**$Cu(acac)_2$[a]**	Sigma-Aldrich	97%	0.404	[6]
**$Fe(acac)_2$[a]**	Sigma-Aldrich	99.90%	0.354	[7]
**$SnCl_4$.5 H_2O**	Sigma-Aldrich	98%	0.200	[8]
**$PdCl_2$**	Sigma-Aldrich	5 wt% in 10 wt% HCl	158	[9]
**$TiCl_4$**	Sigma-Aldrich	ReagentPlus®, trace metals basis, 99.5%	0.285	[10]
**$Ti(BuO)_4$**	Sigma-Aldrich	purum 97.0	0.158	[11]
**TiO_2 anatase**	Sigma-Aldrich	Trace metal basis, 99.7%	2.44	[12]
**P25**	Thermo Fischer	99.50%	0.252	[13]
**Melamine**	Sigma-Aldrich	99%	0.0286	[14]

**NH_4Cl**	Sigma-Aldrich	ACS reagent, 99.5%	0.0752	[15]

[a] with acac standing for acetyl acetonate.

References

(1) Dingenen, F.; Blommaerts, N.; Van Hal, M.; Borah, R.; Arenas-Esteban, D.; Lenaerts, S.; Bals, S.; Verbruggen, S. W. Layer-by-Layer-Stabilized Plasmonic Gold-Silver Nanoparticles on TiO2: Towards Stable Solar Active Photocatalysts. *Nanomaterials* **2021**, *11* (10), 2624. https://doi.org/10.3390/nano11102624.

(2) Stejskal, J.; Kratochvíl, P.; Radhakrishnan, N. Polyaniline Dispersions 2. UV—Vis Absorption Spectra. *Synth. Met.* **1993**, *61* (3), 225–231. https://doi.org/https://doi.org/10.1016/0379-6779(93)91266-5.

(3) Merck KGaA. *Silver nitrate*. https://www.sigmaaldrich.com/BE/en/substance/169877761888 (accessed 2023-08-07).

(4) Merck KGaA. *Gold(III) chloride trihydrate*. https://www.sigmaaldrich.com/BE/en/substance/goldiiichloridetrihydrate3938316961254 (accessed 2023-08-07).

(5) Merck KGaA. *Chloroplatinic acid hexahydrate*. https://www.sigmaaldrich.com/BE/en/substance/chloroplatinicacidhexahydrate5179018497137 (accessed 2023-08-07).

(6) Merck KGaA. *Copper(II) acetylacetonate*. https://www.sigmaaldrich.com/BE/en/substance/copperiiacetylacetonate2617613395169.

(7) Merck KGaA. *Iron(III) acetylacetonate*. https://www.sigmaaldrich.com/BE/en/substance/ironiiiacetylacetonate3531714024181 (accessed 2023-08-08).

(8) Merck KGaA. *Tin(IV) chloride pentahydrate*. https://www.sigmaaldrich.com/BE/en/product/sigald/244678 (accessed 2023-08-08).

(9) Merck KGaA. *Palladium(II) chloride solution*. https://www.sigmaaldrich.com/BE/en/product/aldrich/283606 (accessed 2023-08-08).

(10) Merck KGaA. *Titanium(IV) chloride*. https://www.sigmaaldrich.com/BE/en/product/sigald/208566 (accessed 2023-08-08).

(11) Merck KGaA. *Titanium(IV) butoxide*. https://www.sigmaaldrich.com/BE/en/product/sial/86910 (accessed 2023-08-08).

(12) Merck KGaA. *Titanium(IV) oxide, anatase*. https://www.sigmaaldrich.com/BE/en/product/aldrich/637254 (accessed 2023-08-08).

(13) Thermo Fischer Scientific. *Titanium(IV) oxide, Aeroxide(R) P25, Thermo Scientific Chemicals*. https://www.thermofisher.com/order/catalog/product/384290010 (accessed 2023-08-08).

(14) Merck KGaA. *Melamine*. https://www.sigmaaldrich.com/BE/en/substance/melamine12612108781 (accessed 2023-08-08).

(15) Merck KGaA. *Ammonium chloride*. https://www.sigmaaldrich.com/BE/en/substance/ammoniumchloride534912125029 (accessed 2023-08-08).
